# Advancing crop improvement through GWAS and beyond in mung bean

**DOI:** 10.3389/fpls.2024.1436532

**Published:** 2024-12-18

**Authors:** Syed Riaz Ahmed, Muhammad Jawad Asghar, Amjad Hameed, Maria Ghaffar, Muhammad Shahid

**Affiliations:** ^1^ Nuclear Institute for Agriculture and Biology College, Pakistan Institute of Engineering and Applied Science (NIAB-C, PIEAS), Faisalabad, Pakistan; ^2^ Plant Breeding and Genetics Division, Mung Bean and Lentil Group, Nuclear Institute for Agriculture and Biology, Faisalabad, Pakistan; ^3^ Plant Breeding and Genetics Division, Marker Assisted Breeding Group, Nuclear Institute for Agriculture and Biology, Faisalabad, Pakistan

**Keywords:** QTLs, mung bean, GWAS, high-throughput phenotyping, structural variants

## Abstract

Accessing the underlying genetics of complex traits, especially in small grain pulses is an important breeding objective for crop improvement. Genome-wide association studies (GWAS) analyze thousands of genetic variants across several genomes to identify links with specific traits. This approach has discovered many strong associations between genes and traits, and the number of associated variants is expected to continue to increase as GWAS sample sizes increase. GWAS has a range of applications like understanding the genetic architecture associated with phenotype, estimating genetic correlation and heritability, developing genetic maps based on novel identified quantitative trait loci (QTLs)/genes, and developing hypotheses related to specific traits in the next generation. So far, several causative alleles have been identified using GWAS which had not been previously detected using QTL mapping. GWAS has already been successfully applied in mung bean (*Vigna radiata*) to identify SNPs/alleles that are used in breeding programs for enhancing yield and improvement against biotic and abiotic factors. In this review, we summarize the recently used advanced genetic tools, the concept of GWAS and its improvement in combination with structural variants, the significance of combining high-throughput phenotyping and genome editing with GWAS, and also highlights the genetic discoveries made with GWAS. Overall, this review explains the significance of GWAS with other advanced tools in the future, concluding with an overview of the current and future applications of GWAS with some recommendations.

## Graphical Abstract

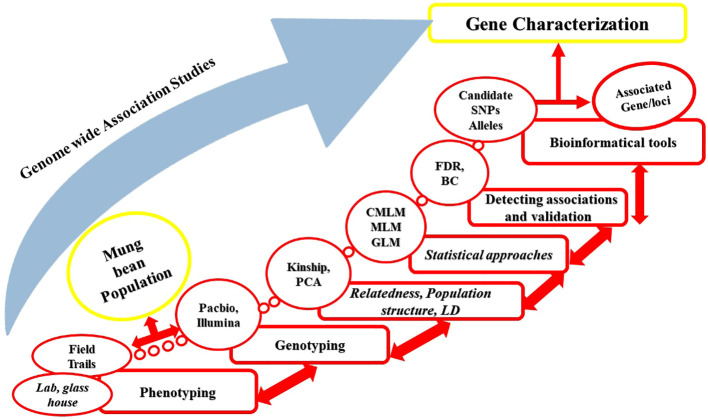

## 1 Introduction

Mung bean (*Vigna radiata* L.) is an important food and cash crop in the rice-wheat-based farming systems of Southeast and South Asia and is also cultivated in other regions of the world, especially in the warm regions of the United States, Canada, Australia, and dry parts of southern Europe. Mung bean is native to the Indo-Burma region of Asia, probably first domesticated there, and is believed to have originated in the subcontinent gene center. The wild ancestors of mung bean, *V. radiata* var. *sublobata*, are also from India and can be found in the sub-Himalayan tract, in the Tarai region and in various parts of eastern and western India. Subcontinent is the main center of mung bean diversity, which spreads across the continent from the Himalayas in the north to the southern peninsula and northeastern regions (Mishra et al., 2022). The Indo-Gangetic plains are considered a secondary center of diversity for mung bean. In the past, mung bean seeds were taken by traders and emigrants from Asia to the parts of South America, Latin America, East Africa, Middle East, and Australia (Manjunatha et al., 2023). The area under mung bean cultivation is increasing worldwide and the reasons behind this are its tolerance to heat and drought stresses, low input requirements, high nutritious profile, and most importantly the short crop duration (70 days). Therefore, mung bean has become the most popular niche crop to fill the time gap between wheat (after harvesting) and rice (before sowing). Mung beans thrive in the humid and hot climates of tropical and subtropical regions. They need an annual rainfall of 600 to 870 mm. The best temperature for mung bean growth and development is between 28 and 30°C, though it can tolerate temperatures up to 45°C. The crop is susceptible to waterlogging but can handle slightly salty soils. Mung beans grow well in well-drained loamy to sandy loamy soils with a pH range of 5 to 8 (Sosiawan et al., 2021). Currently, it is cultivated in over six million hectares (6m ha) worldwide which is about 8.5% of the global pulse area and therefore has become one of the most important edible legume crops (Hou et al., 2019). However, the yield of mung bean in some countries is still very low, ranging from 0.5 to 1.5 t/ha (Hou et al., 2019).

Mung bean is being consumed throughout the world in different forms. The seeds of mung bean are rich sources of proteins, minerals (such as potassium, magnesium and iron), vitamins and dietary fiber compared to other legumes. On dry weight basis the seed of mung bean comprised of 62 to 65% carbohydrates, 3.5 to 4.5% fiber, 4.5 to 5.5% ash, 1 to 1.5% oil and 24 to 28% proteins (Azmah et al., 2023). The proteins of mung bean comprise all the essential amino acids such as lysine, arginine, methionine, tryptophan, isoleucine, valine, phenylalanine, and leucine (Zhang et al., 2024). During sprouting, it has been observed that the proteolytic cleavage of vitamins, amino acids, minerals, and proteins is significantly high. Mung bean holds significant importance in vegetarian diets due to its large and easily digestible proteins. Therefore, mung bean consumption along with other cereals is increasing in the daily human diet (Sehrawat et al., 2024). Mung bean regular consumption not only helps in managing body weight but also provides antioxidant properties, improves digestion, and reduces cholesterol levels in the body to reduce or prevent the risk of chronic diseases. Besides, its nutritious profile, mung bean also plays a significant role in improving soil structure and fertility through nitrogen fixation (Ahmed et al., 2023).

Due to its agronomic and economic importance, it has been used as a model crop to study genomic and genetics studies in other crops of the *Vigna* group. Mung bean is a diploid (2n) in nature with 22 chromosomes and a small genome of around 579 Mb (Somta et al., 2022). In the last few years, research for mung bean has widely expanded since its full genome was sequenced by (Kang et al., 2014). However, its genome has not yet been explored in the ways other models and agronomic crops like *Arabidopsis thaliana*, rice, wheat, cotton, and maize have been explored. Since mung bean has about 14,187 accessions in the central genebank (the second largest collection in genebank after soybean), it provides an excellent resource to efficiently exploit genetic resources in improving future breeding programs (Schreinemachers et al., 2014). Comparing the re-sequenced genes with the reference genome to check the genetic variations and molecular basis can help in understanding mung bean adaptation to different biotic and abiotic stresses. Moreover, unlike other crop species, the cross compatibility among *Vigna* species has not been widely explored or understood, and so their gene pool. However, there is generally no barrier to cross-compatibility between domesticated cultivars and their closes relatives. Some studies have explored wide hybridization to expand the genetic base of *Vigna radiata* using *V. trilobata*, *Vigna umbellata*, and *Vigna mungo*, showing that interspecific barriers can be easily overcome (Lin et al., 2023). Few studies have classified the gene pool of mung bean GP-1, GP-2 and GP-3. The GP-1 consist of *Vigna radiata* and *Vigna sublobata*. The GP-2 consist of *Vigna mungo*, *Vigna umbellate*, *Vigna trinervia*, *Vigna tenuicaulis*, *Vigna stipulacea*, *Vigna grandiflora* and *Vigna subramaniana*. The GP-3 consist of *Vigna angularis* and *Vigna aconitifolia*. Crop improvement has always been the priority of plant breeders (Gayacharan et al., 2020). Crop betterment mainly depends on the availability of genetic variability, which can be found naturally (wild relatives) or induced artificially through hybridization or mutagen. Phenotypic variations within plant species including mung bean are due to the spontaneous natural genetic mutations that are maintained in nature by natural selection, artificial and evolutionary processes. Natural variations have brought great advances in understanding plant physiology, morphology, and its response to adverse climatic conditions. The importance of genetic variation in crop can be understood by elucidating the genetic modifications in agronomic and yield-related traits. For example, pod shattering in mung bean (one of the major issues causing substantial yield loss) is controlled by two quantitative trait loci (QTL) regions (LG1 and LG7). LG7 has also been reported in azuki bean but LG1 is specific in mung bean. Pod shattering in mung bean has been improved through domestication by inducing genetic variation which increased grain yield. Vairam et al.(2017) also reported the improvement of pod shattering in two mung bean genotypes (NM 65 and CO-Gg-7) through induced mutation (Ethyl methane sulphonate and gamma rays) in M2 and M3 generations (Vairam et al., 2017). Genebanks provide a wide source of genetic variation which has been widely used in improving plant species via introducing desired alleles for enhancing yield and developing resistance against biotic and abiotic stresses. On the other hand, modern breeding techniques and domestication processes have also resulted in narrowing down the genetic variation in cultivars that limit crop yield and adaptation.

The last two decades have witnessed tremendous computational and technological advances in nucleic acid sequencing. These advances in the field of genome sequencing are due to the simultaneous sequencing of multiple DNA molecules at a high-speed rate and low sequencing cost (Mardis, 2017). Recently, Miga et al. (2020) for the first time presented the gapless telomere-to-telomere fully sequence assembly of the human X chromosome; before this, thousands of unresolved gaps persisted and no single chromosome was sequenced end to end in any organism. Now, these advances in sequencing technologies have made the genetic improvement of significant traits in mung beans (e.g., early maturity, resistance to mung bean yellow mosaic virus, pod shattering, and seed size) possible. High-throughput-sequencing (HTS) or Next-generation-sequencing (NGS) techniques like genotyping-by-sequencing (GBS) offer the possibility to study thousands of single nucleotide polymorphisms (SNPs) that are associated with the important traits of mung beans. Besides advances in sequencing technologies, numerous excellent statistical-based genetic methods such as whole-genome sequencing (WGS), whole-exome-sequencing (WES) and Genome-wide-association-studies (GWAS) have been proposed to identify genes or alleles controlling target traits. GWAS is a useful technique that can successfully identify the genes of interest for many traits in mung beans as it is based on phenotype and genotype association. In this review we discuss in detail the advancements in GWAS overcoming its limitations, the current status of GWAS in mung bean, discoveries of *k-mers* and structural variations (SVs) as new markers, the status concerning integrating GWAS and high throughput phenotyping in plants (a step forward in unlocking other levels of molecular breeding), expounding the loci found through the multi-scale plant traits obtained by different high-throughput phenotyping techniques in GWAS. In our review, we have focused on mung bean studies as an excellent example of a model pulse crop that has significant genetic improvement due to the identification/discovery of useful novel genes and QTLs, used as markers during selection processes with GWAS. The inherent challenges and future directions are also discussed to enhance our understanding of GWAS, PWAS, and HTP with some guidance for future research.

## 2 Genome-wide association studies

GWAS detects hundreds of thousands to millions of genetic variants (single nucleotide polymorphism-SNPs) across the genomes of many individuals to identify significant associations between phenotype and genotype. GWAS has revolutionized the field of genetics, especially dealing with complex traits over the past decade. GWAS greatly facilitates analyzing the genetic architectures associated with complex traits and thoroughly explores the genetic basis of phenotypic diversity.

**Figure 1:**
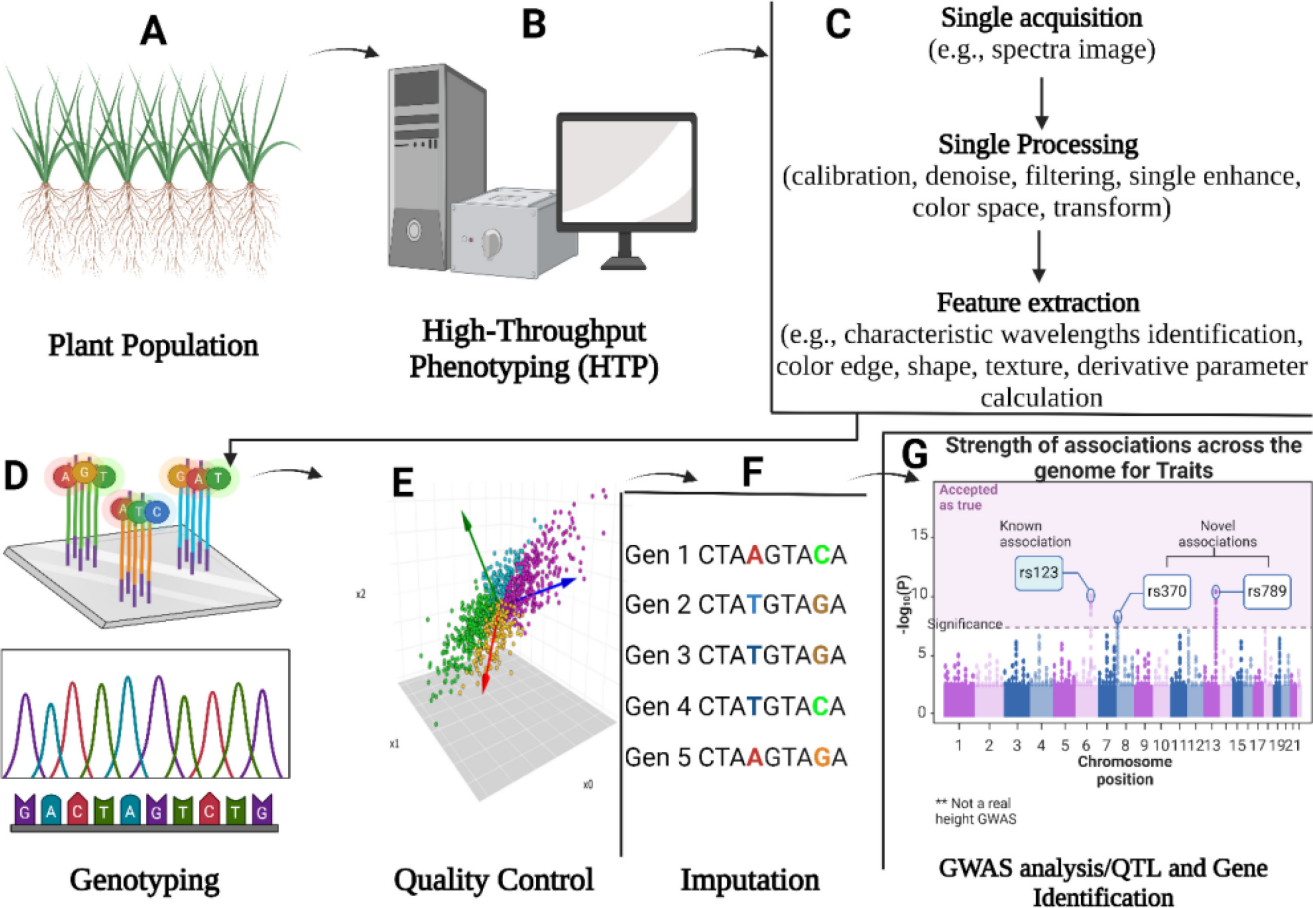
**(A)** Selection of plant population based on the research objective. The plant population should support the hypothesis before the experiment such as if the trait of interest is plant height, then the population be variation for plant height. **(B)** Phenotypic data should be carefully collected from the targeted plant population. To avoid or minimize human errors during data collection, advanced high-throughput phenotyping tools must be used to collect data. **(C)** Advanced high-throughput phenotyping processing unit (combinations of different tools like camera and picture analysis software). **(D)** Genotyping refers to collecting genotypic data using advanced sequencing tools such as WES, WGS, and NGS. **(E)** Quality control involves different steps with wet laboratory work like DNA switches and genotyping calling and dry laboratory work like SNPs calling, principal components analysis (PCA), and population strata detection. **(F)** Detection of the causative or trait associated SNPs across different individuals using reference genome alignment, enhancing the resolution and completeness of genotypic data. The SNPs are represented in different colors (red, blue, green, yellow) to indicate varying physical distances from the causal mutation and to illustrate linkage disequilibrium (LD) decay patterns, where SNPs closer to the causal mutation may exhibit complete LD. **(G)** Using an appropriate model for testing genetic associations for each genetic variant, identification of the QTLs, INDELS, and SNPs associated with a trait of interest.

Unlike GWAS in humans, GWAS in plants uses a permanent resource, a population of diverse genotypes that can be re-phenotyped for several traits and only needs to be genotyped once and one can subsequently generate specific mapping populations for particular traits or QTLs (Huang and Han, 2014). The basic theme of GWAS is to compute the association between markers and phenotypes of interest from a diverse panel. The effectiveness and robustness of GWAS in dissecting quantitative traits in crops including mung bean has been fully demonstrated and, is expected to be more effective in identifying the causative gene/loci(s) for complex traits by utilizing recently available large population and high-throughput sequencing technologies. A large number of alleles (detected through GWAS) and historical recombination events can be used to generate a high-resolution genetic map (Rafalski, 2010) (**Figure 1**). In association mapping populations, historical-recombination events that assembled through several generations with the help of historical Linkage Disequilibrium (LD) which persist among the representative accessions and enhance association analysis resolution via rapid LD decay (Jaiswal et al., 2019).

**Figure 2:**
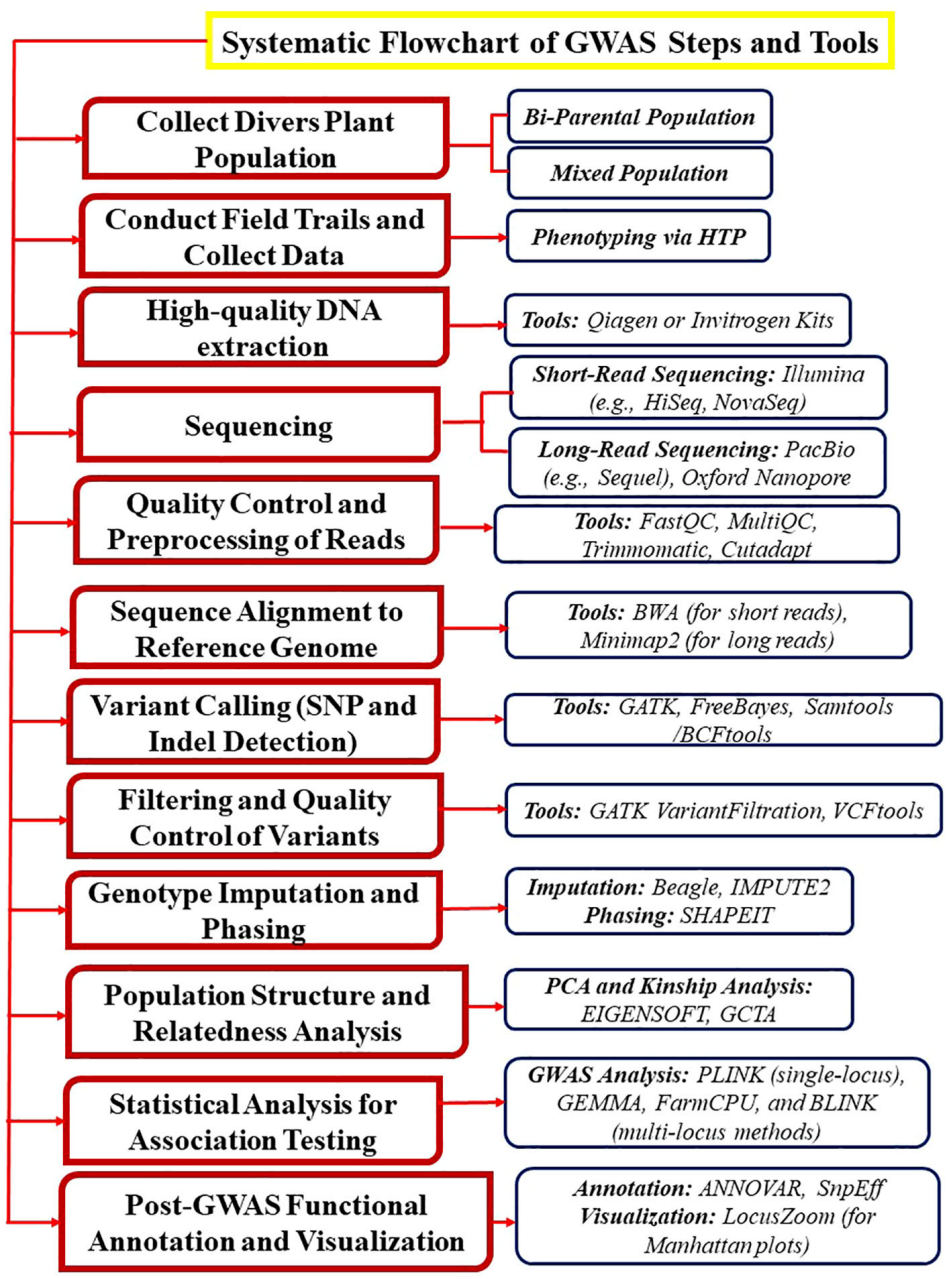
This illustration explains the steps and tools involved in performing GWAS in mung bean and other crops. The process begins with the collection of a genetically diverse plant population (e.g., bi-parental or mixed populations). Next, field trials are conducted, and phenotypic data for traits of interest is collected using high-throughput phenotyping (HTP) techniques. High-quality DNA is then extracted using Invitrogen kits, followed by sequencing with advanced platforms such as PacBio. Finally, various analytical tools are applied to identify the associated SNPs.

GWAS maps quantitative traits and dissect natural genetic variation in combination with genotyping platforms in different crops including mung bean. For example, In GWAS analysis, the use of gene-based 9k SNPs Illumina™ chip provides a higher-genetic resolution that helps in identifying new alleles that improve crop quality, adaptation, and productivity (Thabet et al., 2021) (**Figure 2**). In mung bean, GWAS will be more informative and robust if we use the newly generated 50k Illumina Infinium iSelect genotyping array. The primary objective of conducting GWAS is to identify causal factors for a given trait and determine the genetic architecture of a specific trait. Crop traits can have either simple genetic architecture (controlled by a low number of loci e.g., mung bean seed color) or complex genetic architecture (controlled by a large number of loci e.g., mung bean lobed leaflets).

Several steps have been taken so far to improve GWAS methodology but some factors still exist that limit the power of GWAS.

### 2.1 Factors limiting GWAS power

Many factors limit GWAS’s power to detect true associations between phenotype and genotype. Some of the factors are described below:

#### 2.1.1 Variation in phenotypic data

The raw phenotypic data should be carefully analyzed with outliers identified before performing GWAS. The high level of variation in the data from normal variation data points can limit the power of GWAS and might result in false positive or false negative associations. If there are outliers in the phenotype data, the next step should be to assess the impact of these outliers on the GWAS. The boxplot is used to test the effect of outliers and visualize the data and if there are extreme outliers in the data they should be excluded. While performing all these steps and removing outliers, it should be highlighted that the removal of outliers should not affect the phenotypic variance as it is very important for association. Additionally, once the filtration of data is completed, traits with high or moderate heritability must be considered for GWAS because heritability is one of the great indicators of how strong the phenotype is associated with genotype and how much the genetic variance has been contributed to phenotype. The power of GWAS to detect true associations among phenotype and genotype is also affected by low broad-sense heritability.

#### 2.1.2 Total number of individuals in the whole population

Population size or sample size is considered a key factor while performing GWAS as obtaining meaningful results is completely dependent on the sample size. Population size is important for explaining portions of genotypic and phenotypic variance; therefore, an increase in sample size will enhance the chances of having true associations, overcoming rare variants, and an acceptable frequency within the population. Sample size ranging from 100 to 500 (or > 500) individuals is needed or acceptable for performing GWAS and the sample size below 100 is considered as a disadvantage that reduces or limits the power of GWAS. Selection of the individuals from a large population for GWAS may be based on the researcher or breeder’s trait of interest, genetic background, growth habit, biological status, and geographic region or location. Mostly, the variation among the individuals within a population can be accessed through phenotypic observation but genotypic information can also be used to access the genetic variation. If the extensive genetic information of individuals is not available, even though their genetic diversity can be estimated through genetic markers (DNA markers) for some of the important traits such as plant height, clusters per plant, pods per cluster, early and late maturity and photoperiod response like in case of mung bean. Once the genotypic and phenotypic analyses of individuals are completed, the individuals with maximum variation are selected for the study. This careful selection of the individuals from the population can detect novel true associations due to greater genetic variation that can be utilized in different aspects of future breeding programs.

#### 2.1.3 Population structure

Population structure is one of the most important components of GWAS. It is a statistical approach/method that calculates or infers the relationship between individuals within a population. It is essential to consider the genealogical or historical relationship between individuals as it affects the analysis and interpretation of results. Since not all individuals are equally related to one another at a genetic level, this is considered the major limitation of GWAS. If the population structure is ignored during performing GWAS or not corrected, it results in spurious associations between the phenotype and genotype. STRUCTURE, a computational-based software (freely available, latest version V. 2.3.4) that is used to describe or address the population structure by generating clusters (subpopulation) within a population (also called Q-matrix) to estimate which individual belongs to which subpopulation. STRUCTURE uses multi-loci data of the genotypes and generates highly accurate clusters to describe population structure. Controlling population structure is always the biggest challenge to be tackled properly. Most of the time, the structured associations are removed to control population structure because of limitations in explaining the total number of clusters and assigning each individual to each cluster but that is not always the adequate way. Moreover, structure analyses are always time-consuming and require rigorous computational analysis. Price et al. (2006) introduced another statistical method (called EIGENSTRAT) for addressing or controlling population structure through principal component analysis (PCA) by reducing the dimensional genotype data (Price et al., 2006). The EIGENSTRAT approach uses genotypic data to estimate genetic variations which are described via a small number of dimensions. Yu et al. (2006) introduced the mixed-model technique for controlling spurious associations by considering multiple/several levels of relatedness through a pair-wise relatedness matrix (also known as the Kinship matrix donated by K) (Yu et al., 2006). The kinship matrix uses the genetic information of individuals to calculate or estimate the relationship or relatedness between a pair of individuals. If the value for the relationship between the individuals is high, it means that there is a high genetic similarity between these individuals. For example, individuals from the same geographical regions will have the same level of tendency and therefore be clustered in a similar group. The majority of studies conducted so far in mung bean and other crops have used both PCA and STRUCTURE approaches to validate their results (Sokolkova et al., 2020; Wu et al., 2020a; Reddy et al., 2021; Abou-Khater et al., 2022). Sometimes ADMIXTURE software is also used. PCA represents results in a scatter plot by estimating the total variation among the individuals based on their genetic information. If genotypes are randomly distributed within a plot and generate no group, it means that the population has no population structure. STRUCTURE software plots subpopulation against delta *k* to determine the population structure. STRUCTURE HARVEST is an online website that is used to compress and upload the output results file of STRUCTURE. This software not only provides the acquired population information but also the best *k* for the proposed population. **Table 1** outlines the list of software used in GWAS. Below is the link to STRUCTURE HARVEST

**Table 1:** List of recently developed efficient software for GWAS and genetic analysis.

Software/programs/tools	Application/use	Reference
SMR	Figure out whether the trait and SNP associations are mediated by gene expression levels using Mendelian randomization approach	
Mendelian randomization	Evaluation of causal relation among traits based on genetic overlap utilizing statistics summary of GWAS as input file	(Burgess et al., 2015)
PLINK/PLINK2	Use in different steps while performing GWAS, especially in quality control such as filtering SNPs to separate the associated SNPs from bad SNPs using Hardy Weinberg equation, minor allelic frequency, and genotyping call rate.	(Purcell et al., 2007)
MACH/Minimac	Use to impute missing genotypes adjacent to an available reference panel matched for ancestry and Minimac involved in speeding imputation time.	(Scott et al., 2007)
BEAGLE	Use to impute missing genotypes adjacent to an available reference panel matched for ancestry	(Browning et al., 2018)
GATK	Use for selection of indels and SNPs; acquire reference genome as input file	(Liu et al., 2022b)
IMPUTE_2_	Use to impute missing genotypes adjacent to an available reference panel matched for ancestry; implement more memory when compared to other tools used for imputation	(Howie et al., 2011)
RICOPILI	Use for quality control of raw genetic data and in meta-analysis it requires statistics summary as input file	(Lam et al., 2020)
PLINK	Use to filter the SNPs to minimize the chances of error and identify the real associated SNPs, mostly used after using GATK for further filtration of SNPs	(Han et al., 2022)
BWA-MEM	Use to map reads to the assembled sequence	(Liu et al., 2022a)
SMART-PCA	Use for raw genotypic/sequencing data PCA; provides PCA at the individual level that helps in correcting population stratification	(Kinnersley et al., 2015)
Hisat2	Use to read mapped clean reads to reference genome file	(Liu et al., 2022b)
FastGWA	Used for mixed model genetic association analysis	(Jiang et al., 2019)
BGENIE	Use for continuous phenotypes genetic association: analyses extremely large sample size than is > 100,000; custom made for UK Biobank BGENv1.2 file format	(Bycroft et al., 2018)
SNPTEST	Use for testing SNPs or genetics associations, perform well with IMPUTE_2_	(Band and Marchini, 2018)
Softonic	Use for statistical data analysis and mostly for principal component analysis (https://origin-1.en.softonic.com/)	(Liu et al., 2022b)
FlashPCA	Similar to SMART-PCA but faster and more scalable with increasing sample sizes compared to SMART-PCA	(Abraham et al., 2017)
PowerMarker		
BamTools/FreeBayes variant caller	Use to call SNPs from raw sequencing or fine genotyping data using reference genome panel (https://github.com/ekg/freebayes)	(Rajendran et al., 2021)
PrediXcan	Using GWAS statistical summary as input file to Prioritize likely causal genes based on transcription data	(Gamazon et al., 2015)
STRUCTURE	Use for structure analysis in GWAS population	(Han et al., 2022)
KMC	Use to estimate the distribution of K-mers across the genome with different parameters	(Liu et al., 2022a)
GenomeScope	Use to estimate genome size, acquire GWAS raw sequencing file as input	(Liu et al., 2022a)
REGENIE	Use for analyzing a large population (>100,000) genetic association and has the ability to assess multiple phenotypes at once; memory effective and rapid	(Mbatchou et al., 2021)
QTL Tools	Use for QTLs identification and analysis; required raw genomic sequenced data as input	(Delaneau et al., 2017)
LDSC	Partitioned SNP-based heritability analyses showing enrichment in sets of functionally related SNPs	(Bulik-Sullivan et al., 2015)
DEPICT	Use predicted gene functions to assess enriched pathways and systematic prioritization of genes	(Pers et al., 2015)
Power Marker/SNPhylo	Uses SNP data to develop an un-rooted phylogenic tree	(Reddy et al., 2021; Sandhu and Singh, 2021)
MAGMA	Use regression framework with competitive testing to assess gene-set and gene-based analysis; permits custom gene sets testing including s options for conditional and interaction testing between gene sets	(De Leeuw et al., 2015)
LDPred-2/LD Pred/PRScs/SBayesR	Estimation of posterior effect sizes of SNPs using a Bayesian shrinkage approach	(Vilhjálmsson et al., 2015; Privé et al., 2020)
VCFtools	Use to identify chromosomal regions possessing high genetic differences or maximum nucleotide diversity among subpopulations	(Han et al., 2022)
GenomicSEM	Use to assess multivariate genetic correlation using GWAS-based summary statistic	(Grotzinger et al., 2019)
LAVA	Use to assess local multivariate genetic correlation using GWAS-based summary statistic	(Werme et al., 2021)
p-HESS	Use to assess local SNP-based heredity and genetic correlation using GWAS-based summary statistic	(Shi et al., 2017)
superGNOVA	Use to assess local genetic correlation using GWAS-based summary statistic	(Zhang et al., 2020)
fastPHASE	Use to detect SNP markers with MAF 0.05 (http://stephenslab.uchicago.edu/software.html)	(García-Fernández et al., 2021)
SumHer	Use to assess genetic correlation between phenotypes using summary statistic as input; possess several other functions too including assessment of selection bias and partitioned SNP-based heritability	(Speed and Balding, 2019)
GCTA	Use to assess the genetic correlation between phenotypes using raw sequencing file as input	(Yang et al., 2011)
BLUP	Use for different tasks in GWAS such as statistical analysis, association mapping, etc.	(Sandhu and Singh, 2021; Abou-Khater et al., 2022)
FUMA	Use for functional annotation of transcriptomics, proteomics, genomics, and also regulatory regions such as chromatin interaction information and integrates and visualizes all output	(Watanabe et al., 2017)
ANNOVAR and VEP	Use for functional annotation of transcriptomics, proteomics, genomics, and also regulatory regions	(Mclaren et al., 2016)
HaplotypeCaller	Use to identify potential variants in individual samples and generate results in the GVCF file	(Han et al., 2022)
METAL	Use GWAS statistics summary file as input for weighted meta-analysis	(Willer et al., 2010)
GWAMA	Use for Fixed and random effects meta-analysis; allows the specification of different genetic models	(Mägi and Morris, 2010)
FINEMAP	Use to calculate effect sizes and heritability owing to likely causal SNPs; draw statistical-fine mapping acquiring GWAS summary statistics as input file	(Benner et al., 2016)
SuSIE	Use GWAS statistical summary for fine mapping and LD information from a reference panel; based on a Bayesian modification of a forward selection model	(Wallace, 2021)
PAINTOR	Use GWAS statistical summary for fine mapping and functional genomics data for prioritizing likely causal variants	(Kichaev et al., 2014)
GAPIT	Use to perform statistical analysis such as PCA and also develop genetic kinship matrix performing GWAS	(Gela et al., 2021)

(https://taylor0.biology.ucla.edu/structureHarvester/).

#### 2.1.4 Distribution of allelic frequency

Another important component that limits GWAS power is the distribution of allelic frequency; as only a few alleles/loci are present in a few individuals against the whole population. If the number of alleles is fewer or rare, it results in low-resolution power. Thus, allele frequency analysis and distribution directly affect the phenotypic and genotypic associations. If functional alleles are present in the population with low frequency, their detection becomes very challenging unless they have a major effect on the phenotype. If one ignores allelic frequency during GWAS, this might lead to false results. The majority of studies in GWAS focus entirely on common/rare variants and mostly display the allelic frequency at >5%. It means that if the entire population comprises 500 individuals, only 25 individuals are carrying that allele. It shows that this variant is rare with minor allele frequency (MAF) at <5%. This MAF or rare allele explains the variation only in a particular group of individuals within the entire population however, this variant/allele could be important and helpful in future breeding programs. For instance, Youssef et al. (2017) studied a barley population comprised of 209 accessions out of which 13 accessions were collected from East Asia (Youssef et al., 2017). They reported that the 11 accessions from East Asia (out of 13) were carrying the allele (MAF <5%) that significantly affected several complex traits like greater leaf area, number of leaves, and number of tillers. This finding indicates that low-frequency alleles/loci can have immense effects on complex traits. They also proposed that population structure must be carefully studied and linked with GWAS outputs to interpret the results. However, the lower MAF also impacts the ability to detect and utilize the genetic variants associated with the trait of interest. Low MAF also reduces the statistical power to identify the significant association between the traits and alleles. Low MAF increases the chances of false negative results during SNPs association with the trait of interest and thus the reliability of the results gets reduced.

#### 2.1.5 Linkage disequilibrium

In a given population if the alleles are associated non-randomly, this is called linkage disequilibrium. LD is another important factor that needs to be considered carefully during GWAS analysis, particularly when defining intervals of tightly associated SNPs which help in explaining the foremost significant loci. If one ignores the alleles’ non-random association at different loci, then both causative and non-causative alleles will be incorporated during analysis and will result in false associations. LD is very important in finding all the markers acquired for covering or scanning the whole genome by determining the distance among loci with the help of LD. If the value of LD is high it means that a small number of markers are required to cover the whole genome (Semagn et al., 2010; Mathew et al., 2018). Long-range LD enhances the chances of spurious associations therefore calculating LD at the beginning of association analysis is necessary to avoid false/spurious associations. The coefficient of LD can help in measuring the values of how likely two loci are associated and share recombination and mutation history. This analysis is performed using a disequilibrium matrix which displays pair-wise calculations between loci by utilizing the two most common statistics D’ and r^2^ to measure LD (Flint-Garcia et al., 2003). Several LD analyses performed in plants to date have concluded that D’ is likely to be affected by MAF and population size while r^2^ is a strong value for estimating how QTL of interest and loci are correlated. LD is likely to be used for estimating the association values (D’ or r^2^, >0) between loci as it is important to link the causative SNP with phenotypic variation. It is necessary to consider LD within SNPs as well as in causative alleles during statistical analyses because these analyses reveal whether SNPs identified within LD are significantly associated with a phenotype or not. At this stage in such analysis, it is recommended to consider all SNPs above the threshold level (sometimes every single SNP even below the threshold level) to determine which SNP can clearly explain phenotypic variation since not every highly-associated SNP can have a greater impact on phenotype. SNPs within LD having an r^2^ value > 0.2 must be considered for statistical analysis because they might be useful to detect causal loci, especially for those QTLs that are present in the centromeric region (Nadeem et al., 2024).

Mapping resolution (i.e., total markers and density of a given population) in GWAS is of great importance and it is identified through genome size and LD-decay (the rate at which LD declines with physical or genetic distance). The rate of LD decay over a distance (physical/genetic) varies dramatically for loci within a population, within a genome, and among species. To accelerate the rate of LD decay, a greater number of markers would be required for whole-genome association analysis. This LD decay rate helps find the total number of markers required for GWAS by dividing the genome size by the distance at which LD is decayed (Fedoruk, 2013). LD decay in self-pollinated crops such as mung bean is always larger compared with cross-pollinated crops like maize and therefore requires a few markers to cover the whole genome. In mung bean, the LD decay for cultivated and wild species is estimated at about ∼100 and ∼60, respectively (Noble et al., 2018).

If one is interested in estimating the historical recombination events within a particular species then LD pattern analyses within a population can help. However, this depends on several factors like population structure, population size, genotype selection, genetic drift, mutation rating, random mating, recombination rate, and allele frequency. In an association panel (i.e., in artificial selection by researchers), the allelic frequency is not expected to fit with the Hardy-Weinberg principle (HWP) proportion for a given loci (i.e., unlike bi-parental population, genotype frequencies cannot be predicted by association population allele frequencies). However, SNPs that do not fit in HWP are usually excluded from GWAS analysis (Anderson et al., 2019b). In cross-pollinated species, LD decay occurs more rapidly than in self-pollinated species because of large effective recombination. Recombination events in association populations gathered over generations enhance mapping resolution due to a greater number of alleles. If the population size is small, there is a possibility that genetic drift may result in the loss of rare alleles as well as an increase in LD levels. In addition, selection can also increase the level of LD such that if recombination or mutation occurs among neighboring alleles, they will both be under selection pressure. Thus, association population selection can result in alleles that control specific phenotypes (locus-specific linked alleles) which usually appear in LD. Moreover, migration also increases the level of LD in the population and greatly affects the genetic structure of the association panel. Ignoring genetic drift, migration, mutation, and selection could lead to alleles in linkage equilibrium (D’ or r^2^ = 0). Therefore, critical estimation of population structure and identification of subgroups at the beginning of analyses can reduce all these factors.

### 2.2 Newly introduced approaches for improving and enhancing GWAS power

The introduction and improvements of new approaches for GWAS have always been an area of interest since LD-based association mapping was first presented (Lander and Kruglyak, 1995). So far, three major areas have been highlighted with the notion that these will not only overcome the above-mentioned limitations but also improve GWAS in different aspects. The three evolving areas include; (1) the development of new efficient marker systems (recently discovered *k-mers* and structural variants(SVs) for genotyping with emphasis on the use of pan-genomics, (2) continuous development and improvements of software and statistical models for statistical analysis to enhance GWAS resolution, and (3) to minimize errors from phenotypic data by introducing high through-put phenotyping techniques (Gupta, 2021b). Simple sequence repeats (SSR) were the first type of markers used in GWAS followed by haplotypes and SNPs. SNPs are the most common type of markers used in GWAS these days. Recently two new classes of markers, *k-mers* and SVs including chromosomal rearrangements (inversions/translocations), insertions/deletions (InDels), presence/absence variation (PAV), and copy number-variations (CNVs) are receiving attention from scientists because they are becoming valuable resources for GWAS.

#### 2.2.1 Genome and GWAS to pan-genome and PWAS

Advances in next-generation technologies (NGS) have made it possible to score thousands of SNPs in a single genotype from an accession panel of species and compare the genome sequence of each genotype with an available reference genome. However, this method cannot score the entire genetic variation present in the genomes of all genotypes of an accession panel used for GWAS. To overcome this issue, it was decided to take advantage of the available genome sequences of individuals within a species, assemble pan-genomes, and use them for GWAS. Tettelin et al. (2005) assembled the first pan-genome in *Streptococcus agalactiae* followed by the development of pan-genomes in plants, animals, and humans (Bayer et al., 2020). Now these pan-genomes are being used as novel reference genomes for GWAS, like the recent acronym PWAS (pan-genome wide association studies) has also been used for GWAS (Manuweera et al., 2019). The applications of *k-mers* and SVs based on early pan-genome studies discovered two key findings; first, in every species there is about 15 to 40% variable gene content, and second, the genes concerned with *k-mers* and SVs are frequently associated with every type of trait including resistance to abiotic and biotic stresses in crops (Gupta, 2021b). Genomic variations within species are found in both gene content (e.g., PAVs of genes, CNVs distribution across genome, and tandem duplicated genes) and repeated genome portions (e.g., centromere repeats, knob repeats, and transposable elements). This variation has been characterized into three components; core fraction (genomic fraction common to all genotypes within a species), dispensable fraction (which might present in the genome of some genotypes but not in all genotypes) and unique fraction (which is unique to an individual genotype within a species). Till now, several pan-genomic studies have been conducted in different crop plants such as barley (Jayakodi et al., 2020; Wu et al., 2022), wheat (Walkowiak et al., 2020), sorghum (Ruperao et al., 2021), rapeseed (Song et al., 2020, Song et al., 2021), soybean (Li et al., 2014), rice (Zhao et al., 2018a), tomato (Gao et al., 2019), *Brassica oleracea* (Golicz et al., 2016; Bayer et al., 2019), *Brachypodium distachyon* (Gordon et al., 2017) and *Arabidopsis thaliana* (Alonso-Blanco et al., 2016; Van De Weyer et al., 2019). However, no study on pan-genomics in mung bean has been reported yet. There is a need for pan-genomics studies in mung bean to explore the complete genetic variations of some interesting traits such as early maturity and seed size for developing early maturity varieties with large seed size.

#### 2.2.2 Characterization of k-mers and SVs for GWAS

**Figure 3:**
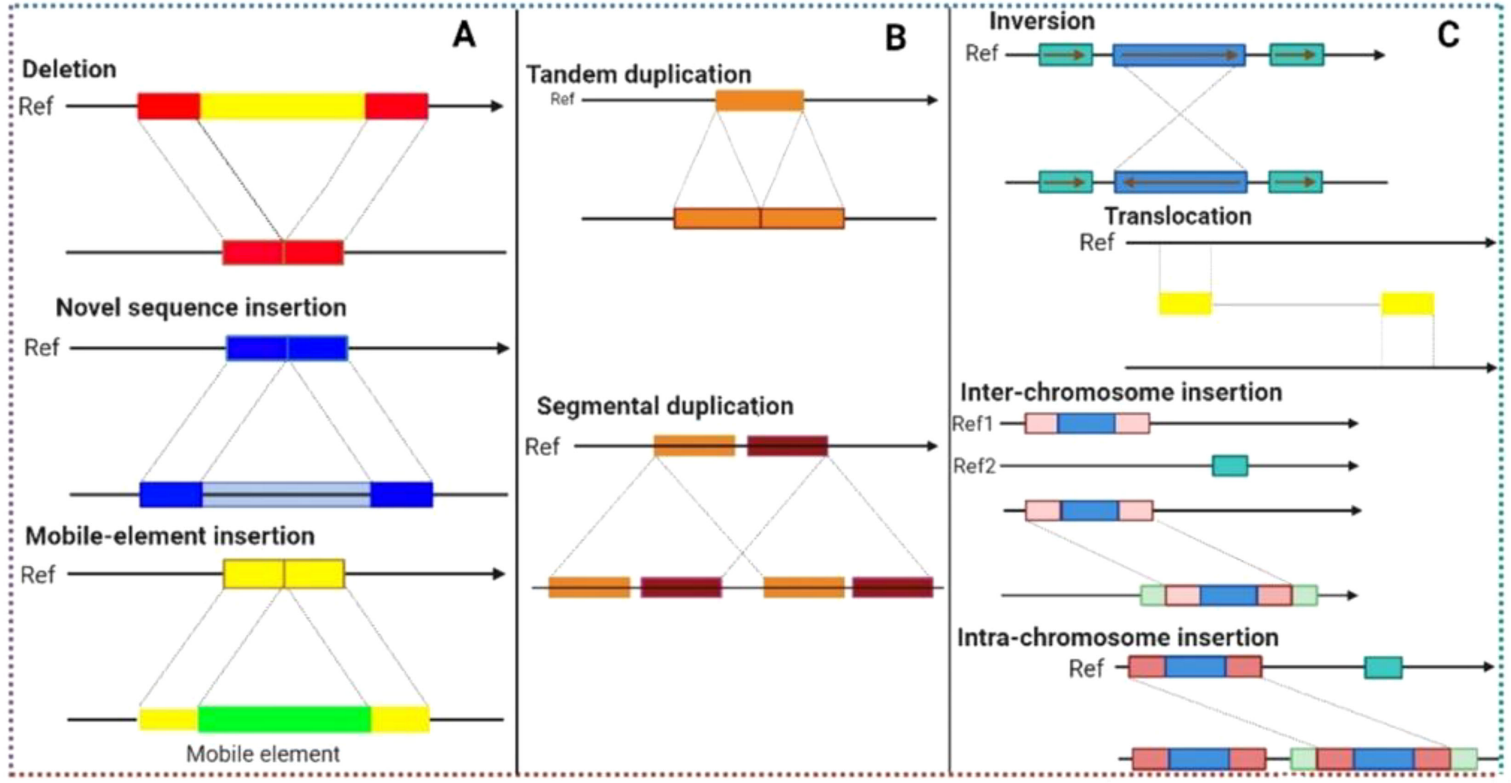
Illustration of different structural variants (SVs) that can be found across crop genomes and responsible for creating genetic variations that lead to genetic diversity. Structural variants (such as deletions, insertions, duplications and inversions) in combination with genome-wide association studies (GWAS) can detect hidden SNPs (associated with traits of interest) that remain undiscovered during GWAS analysis.

During the last few years *k-mers* and Svs have been intensively used for GWAS since pan-genomics have witnessed producing millions of *k-mers* and SVs in single plant species. *K-mer* usually refers to a subsequence in any sequence with a certain length. *K-mers* (they can be in billions to trillions within a species) depend on the *k* value. *k* is the number of nucleotides utilized to develop a set of *k-mers* (**Figure 3**). For example, AGAT is the sequence of four nucleotides present in DNA, so the value of *k* will be (4)*^k^*; therefore, if *k* = 2 then the number of possible *k-mers* is 16, if *k* = 3 then *k-mers* are 64 if *k* = 6 then *k-mers* are 4096 and if the *k* value is 15 or 20 then the *k-mers* will be in billions and trillions, respectively. The value of *k* can be between 2 to 35 or maybe more. *k-mers* with different lengths have already been used for GWAS and pan-genome assembles. *k*-mers are capable of detecting a wide range of polymorphisms without requiring any reference genome and can be used for GWAS. Before *k-mer* utilization in GWAS, deciding on the size of *k-mers* is the first step (Gupta, 2021a). After this, *k-mers* are isolated from short, sequenced reads (acquired from each genotype of an association panel) and then used for *k-mers* genotyping of one or more association panels. *k-mers* genotyping refers to counting each *k-mer* with a particular size (as mentioned above) in each genotype of the association panel. The genotypic and phenotypic data are then used to identify marker-trait associations (MTAs) in the form of *k-mers* just like SNPs. Voichek and Weigel. (2020) expanded the genetic variants detected through GWAS to include major rearrangements, insertions, and detections (Voichek and Weigel, 2020). They directly used raw sequence data files and derived *k-mers* and short sequences as these can mark a huge polymorphism without using a reference genome. Later, they linked *k-mers* associated with phenotype to specific genomic regions. Using this technique, they studied 2000 traits in maize, tomato, and *Arabidopsis thaliana*. Results revealed that MTAs detected through *k-mers* were not different from those detected through SNPs, but *k-mers* allowed detection with more statistical power as compared to SNPs. However, some of the MTAs identified through *k-mers* were not detected earlier using GWAS. They also detected some new associations through SVs and missing regions from reference genomes. This study highlighted the importance of *k-mers* and SVs for GWAS by not only improving GWAS power but also detecting associations with more statistical confidence.

Reduction in sequencing cost of both whole genome sequencing and short reads have allowed characterization of SVs (PAV/CNV) more frequently in crops. PAV and CNV detection techniques have been classified into three categories namely split, pair and depth reads (Alkan et al., 2011). The split read technique involves SVs detection within interrupted short read sequences (Alkan et al., 2011). The read pair technique involves the identification of PAV/CNV based on discrepancies in the distance between paired-end sequences relative to their distance in the reference assembly (Alkan et al., 2011). In the read depth technique, against reference genome short reads are mapped, and the relative depth of a sequence at a locus serves as a proxy for copy number in a particular genotype. Initially, hybridization arrays were used to detect variants but with a greater number of limitations. Later, the availability of whole genome sequencing made the detection of variants much easier but still with some minor limitations. However, these shortcomings have already been addressed to some extent.

Recently, a few other techniques have been developed to further improve the PAV/CNV characterization and also leverage the newly developed library preparation techniques, single-molecules maturation, and long-read sequencing. For instance, connecting molecule approaches such as Strand-Seq, Hi-C, and 10x can retrieve long-range information utilizing short-reads via developing linked reads specialized libraries. Single-molecule techniques (Bionano (optical map) and long read sequencings like Oxford Nanopore and PacBio) permit aligning sequences from several individuals and due to different read lengths; missing sequencings in the reference genome can also be characterized. Both of the above-mentioned techniques have allowed the characterization of both intermediate and small-sized SVs (Levy-Sakin et al., 2019). However, SVs greater than 1Mb can be more effectively characterized through optical maps (Levy-Sakin et al., 2019). SVs with millions of copies in each crop species have already been identified and are intensively being utilized for GWAS/PWAS. Wei et al. (2021) presented a comprehensive quantitative-traits nucleotides [(QTNs) including CNV and PAV] map of rice based on eight GWAS cohorts (Wei et al., 2021). They also developed a genome-navigation system (RiceNavi) for breeding route optimization (BRO) and QTN pyramiding and implemented it in the improvement of Huanghuazhan (intensively grown *indica*rice cultivar). Till now, these developments have led to the most comprehensive characterization of PAV/CNV. Ho et al. (2020) have recently provided a comprehensive review of SVs development in the era of genomics (for more information on SVs read the mentioned review).

## 3 Genetic and molecular advancements in mung bean

**Figure 4:**
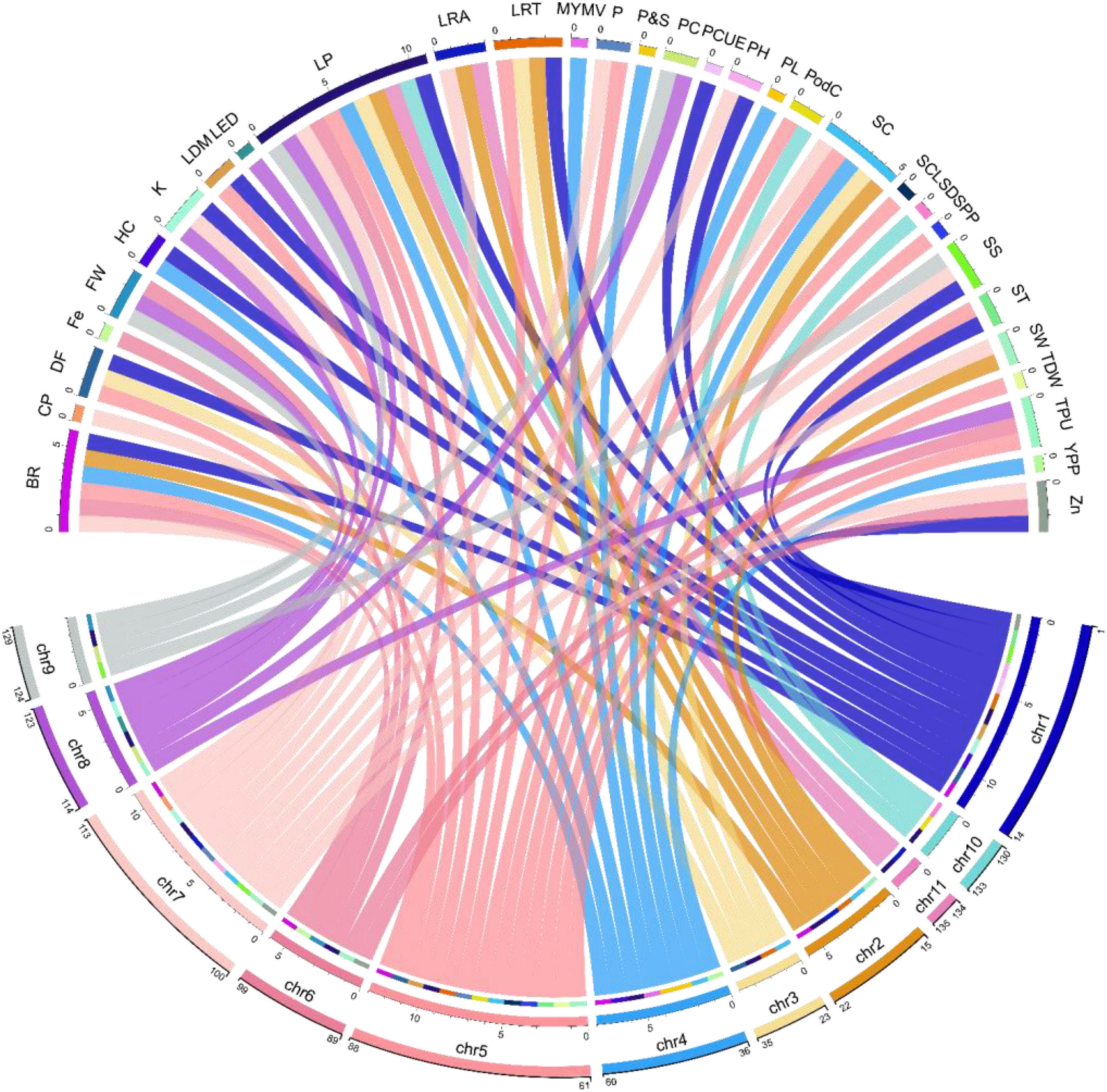
Distribution of some of the most important genes across chromosomes associated with different mung bean traits discovered through GWAS. The numbers on each chromosome (in second line) for example on chr1 (1-14), Chr2 (15-22), Chr3 (23-35) represent the number of genes present on the chromosome associated with the above traits (**Supplementary Table 1**); SC (seed color), BR (bruchid resistance), CP (crude protein), DF (Days to flowering), FW (Fusarium wilt), HC (hypocotyl color), Fe (Iron), LRA (lateral root angle), LDM (leaf drop at maturity), LRT (Leaf related traits), LP (Lectin proteins), LEC (Root length distribution), MYMV (Mung bean yellow mosaic virus), PC (Phosphorus conc.), PCUE (P concentration and P utilization efficiency), P (Phosphorus), PH (Plant height), PC (Pod color), PL (Pod length), K (Potassium), P_S (Quality traits (Protein and starch), SS (Salinity stress), SCL (Seed coat luster), ST (Seed texture), SW (Seed weight), SPP (Seeds per pod), SD (Shoot development), TDW(Total Dry Weight), TPU (Total Phosphorus Uptake), YPP (yield per plant), Zn (Zinc).

Modern genetics, molecular breeding and functional genomics techniques have made plant tolerance against biotic and abiotic stresses easier and faster. Biotic (such as MYMV) and abiotic (drought, salinity and temperature) factors reduce mung bean yield significantly. The emergence and development of the MYMV (through white fly) across India, destroyed the mung bean crop fields completely. Later, this viral disease started spreading rapidly across the borders and started destroying the mung bean crops in other countries like Pakistan and Taiwan. In the early 90s Nuclear Institute for Agriculture and Biology (NIAB), Faisalabad developed the first MYMV-resistant variety through physical mutation (NM-92). The advancement from conventional breeding to mutation breeding (chemical and physical mutagens) was not fast enough as the advancement todays in modern genetics techniques. Till now several crops including mung beans have been improved through modern genetics techniques such as marker-assisted breeding, gene silencing, genome editing, QTLs mapping, and NGS. Understanding the crop’s genetics associated with the traits of interest allows the molecular breeders to identify the loci and construct a genetic map. Subramaniyan and Narayana (2023), developed a mung bean population through crossing TU 68 (resistant male parent to MYMV) and MDU 1 (susceptible female parent to MYMV), to access the mung bean resistance to MYMV through genetic markers. Some of the introgression lines showed significant resistance to MYMV along with high yield. They further identified the genes associated with the disease resistance through using genetic markers. Talakayala et al. (2022), employed CRISPR-Cas at two different locations AV1 (coat protein) and AC1 (rep protein) in mung bean to develop resistance against the MYMV. The transformed lines (containing Cas9 cassette) displayed minimal mosaic symptoms and displayed resistance against MYMV by reducing the accumulation of AV1 and AC1. Besides, several studies have identified many genes in mung bean associated with several biotic and abiotic factors and constructed QTL maps. Some of the examples are given below in details. **Figure 4** contains the several genes identified associated with traits and their chromosomal location in mung bean.

### 3.1 QTLs detection in mung bean through GWAS

We have seen the negative effects of climate change on crop growth and development including a significant reduction in yield. Complex traits like yield and seeds per pod in mung bean are controlled by several alleles and therefore it is difficult to understand the underlying genetic architecture of complex traits (Yuan et al., 2020). For example, GWAS analysis in mung bean recently discovered five QTLs associated with resistance to mung bean yellow mosaic virus (MYMV). The QTLs *qMYMV_10_1_*, *qMYMV_6_1,_ qMYMV_4_1,_ qMYMV_5_1_* and *qMYMV_4_1_* was identified on chromosomes 10, 6, 5, and 4 with a total of 538 SNPs covering 1291.7 cM distance. *qMYMV_4_1_* (on chromosome 4) was found as major and the most stable QTL for resistance to MYMV (Mathivathana et al., 2019). GWAS analyses have discovered several novel QTLs for various traits and environmental conditions like salinity stress in different crops (including mung bean) that have not been reported previously. Salinity stress is known to cause a major yield reduction in mung bean. Liu et al. (2022a) reported seven QTLs (*EVM0012371*, *EVM0002218*, *EVM0029605*, *EVM0033924*, *EVM0022712*, *EVM0017397*, and *EVM0018329*) significantly associated with salt tolerance in mung bean using GEMMA and EMMAX (Liu et al., 2022b). These QTLs are distributed on chromosomes 1 and 3. They also reported that the expression level of candidate gene *VrFR08* was up-regulated under salinity stress. Furthermore, another study reported 5288 SNPs markers through GWAS to mine alleles associated with salinity stress in mung bean. Significantly associated SNPs and QTLs were identified on chromosomes 7 and 9 with 7 and 30 genes, respectively. However, QTL on chromosome 7 stretched from position 2,696,072 to 2,809,200 bp having seven genes but only one gene *Vradi07g01630* was functionally annotated. Similarly, QTL on chromosome 9 stretched from 19,390,227 to 20,321,817 bp having 30 genes but only two genes *Vradi09g09600* and *Vradi09g09510* were functionally annotated (Breria et al., 2020a). Dissecting the root genotypic and phenotypic variability in mung bean accessions using GWAS revealed that chromosomes 2, 6, 7, and 11 possess QTLs that control lateral root angel (LRA), chromosomes 3 and 5 having QTLs that control total dry weight (TDW) and volume (VO) and QTLs on chromosome 8 control total root length growth rate (TRLGR). Moreover, gene description on different chromosomes; chromosome 2 has two genes first (–)-Germacrene D synthase-like and second gene description is not given (both genes are significantly associated with LRA), chromosome 3 has one gene Mannose-1-phosphate guanylyltransferase1 (associated with TRLGR), chromosome 5 has one gene dehydration-responsive element-binding protein 2H (DREB2) associated with TDW, chromosome 6 also has one gene associated with LRA but has no description, chromosome 7 has two genes first Beta-galactosidase 3 and second gene description is not given (both associated with LRA). Chromosome 8 possesses two genes first Monodehydroascorbate reductase, second Uncharacterized LOC106771882 associated with LED. Chromosome 11 has one gene Protein FAR1-RELATED SEQUENCE5 associated with LRA (Chiteri et al., 2022). To this end, several mung bean populations and marker types have been used to study the genetic variability among accessions and a wide range of important traits. For example, the mini core mung bean collection (consisting of 293 to 297 accessions) established by the World Vegetable Centre Taiwan (also called AVRDC) is intensively used for GWAS studies that revealed QTLs for different traits and stress conditions (Breria et al., 2020b; Sokolkova et al., 2020). GWAS output in mung bean provides novel candidate genes and alleles that can be used in future breeding programs to develop resistance to abiotic and biotic stresses and enhance yield to meet targets.

### 3.2 GWAS: a driver of candidate gene discovery in mung bean

Statistical geneticists commonly believe that GWAS have rendered traditional candidate gene identification techniques obsolete (Duncan et al., 2019). The importance of population association mapping in identifying the candidate genes associated with particular traits can be estimated from the number of studies published since 2015. In this section, we shall also illustrate the potential of GWAS in detecting allelic variations with examples shown in **Table 2**. The first genome-wide study in mung bean was conducted by Van et al. (2013) to assess the genetic diversity and identify the SNPs markers associated with resistance the MYMV and seed shattering (Van et al., 2013). They used Illumina Hiseq to sequence Gyeonggi jaerae 5 and Sunhwanokdu (two mung bean cultivars) and sequenced more than 40 billion base pairs (from both cultivars) to a depth of 72x. They identified a total of 305,504 SNPs out of which 42 were significantly associated with both the traits mentioned above. In the beginning, identifying candidate genes using whole-genome sequence data was difficult due to the lack of knowledge of GWAS and the tools/software required for handling the large data. Later Korean scientists, Daovongdeuan et al. (2017) carried out the second GWAS attempt in mung bean to study seed size and color using 218 accessions collected from different regions of the world (Daovongdeuan et al., 2017). They could not identify any significant SNP marker associated with the studied traits at a LOD of 6 and p-value <0.05. This second attempt of GWAS in mung bean once again failed in reporting the candidate genes associated with seed size and color. However, they reported that the studied traits were controlled by several alleles but with minor effects. *VrMYB113* (on chromosome 4) and *Vrsf3′h1* (on chromosome 5) are the first two genes in mung bean discovered using GWAS; that are associated with the seed coat color (Noble et al., 2018) (**Figure 4**, **Supplementary Table 1**). *MYB113* was first reported by Gonzalez et al. (2008) in *Arabidopsis thaliana*, responsible for anthocyanin biosynthesis (Gonzalez et al., 2008). Anthocyanin concentration in mung bean and other plants depends on the expression levels of *MYB113*. miR828 (micro-RNA828) and TAS4 (trans/acting siRNA4) are small endogenous RNAs, responsible for post-transcriptional suppression of *MYB113*.TAS4 and miR828 mutants were developed using CRISPR-Cas to further confirm the involvement of *MYB113* in anthocyanin biosynthesis. The mutant plants accumulated more anthocyanin compared with untreated plants, thus confirming the significant association of *MYB113* with seed coat color (Sunitha and Rock, 2020; Koo and Poethig, 2021). *FRO8* gene is another example detected by GWAS, associated with tolerance to salinity stress in mung bean (Liu et al., 2022b). *FRO8* had a direct connection with the *BELL-1* gene. The BELL-1 like family (BELL) of transcription factors is ubiquitous among plant species and found in regulating a range of developmental processes through interacting with KNOTTED1-like proteins (Kurt and Filiz, 2020). *Jg5489* which is a homolog of *WUSCHEL*-related homeo-box-3 (WUS), associated with yield per plant in mung bean has also been discovered using GWAS. In the same study, they also discovered several other candidate genes *jg35209* and *jg3587* that are homologs to *Glyma09g33350/Glyma09g33340* and *Glyma03g01540* (soybean candidate genes identified using GWAS) associated with days to flowering (Mao et al., 2017).

**Table 2:** List of candidate gene(s) discovered and validated using GWAS in mung bean and other pulses.

Population	Sample Size	Growth habit	Model	Markers	Phenotype	Software/programs/tools	Candidate gene(s)/Gene ID	Chromosome Position	Validation	Reference
Mung bean										
Chinese accessions	112	Summer	GEMMA, EMMA	160.14K	Salinity-stress survival rate 10 and 15 Days	Softonic, Hisat2, and GATK	*VrFRO8*	Chr.1	Comparative genomics, Transcriptome and Metabolomics PCR, statistical analysis, data integrations	(Liu et al., 2022b)
Chinese and other origin accessions	750	Spring and Summer	GEMMA	2.9K	Insect resistance, yield, gain composition, pod width, pod length, flowering period, etc.	KMC, GenomeScope, BUSCO, BWA-MEM, Hi-C, LTR_retriever, RepeatModeler, RepeatMasker, ADMIXTURE, BRAKER2, HISAT2, ProtHint, GUSHR, Infernal, Barrnap, Rfam, r8s, TimeTree, WGD detector, Profiler, MCScan, MaSuRCA, QUAST, CD-HIT, Mosdepth, Picard, EIGENSOFT, iTOL, R-programming, VCFtools and LDBlockShow	jg22573, jg5284, jg13746, jg35209, jg3587, jg30665 and 250+ others.	Chr.1, Chr.4, Chr.5, Chr.7, Chr.10	Markers, Transcriptome and Metabolomics, statistical analysis, data integrations	(Liu et al., 2022a)
USDA and Asian accessions	375	Growth chamber	MLM	26.5K	TDW, VOL, TRL_GR, LED, LRA, etc.	TASSEL	LOC106755829, LOC106753988, LOC106768494, LOC106776541, LOC106772343, LOC106771882, LOC106772343	Chr. 2, Chr. 7, Chr. 11, Chr. 8, Chr. 5	Mapping, Molecular markers, statistical analysis, data integrations	(Chiteri et al., 2022)
Chinese accessions	558	Spring and Summer	GEMMA	69.9K	Branch number, plant height, pod width, pod length, Flowering time, and quality parameters	SAMtools, GATK, e HaplotypeCaller, PLINK, MEGA-X, STRUCTURE, VCFtools, R-programming	Vradi05g00200, Vradi03g06500, Vradi04g07830, Vradi04g07820, Vradi04g07810, radi04g07800	Chr. 5, Chr. 3, Chr. 4	Re-sequencing, variant Mapping, Molecular markers, statistical analysis, data integrations	(Han et al., 2022)
AVRDC accessions	120	Glass house	MLM, CMLM	55.6K	TDW, PC, TPU, PUtE	TASSELv5.0, STRUCTUREv2.3.4, PLINK, MEGA v6.0, PowerMarker v3.51	VRADI01G04370, VRADI05G20860, VRADI06G12490, VRADI08G00070, VRADI08G20910, VRADI09G09030	Chr.1, Chr.5, Chr.6, Chr.8, Chr.9	Sanger sequencing, expression analysis, Markers	(Reddy et al., 2021)
USDA accessions	482	Spring and Summer	CLMM, FarmCPU	264.5K	Qualitative seed traits, 100- seed weight, days to flowering, Plant height, etc.	CLML, GAPIT, BLUPs, FarmCPU, Numericware-i, STRUCTURE, PLINK, SNPhylo, DISTRUCT, R-programming, CLUMPP, adegenet	LOC106774729, LOC106774729, LOC106774729, LOC106756462, LOC106758789, LOC106759308, LOC106760769, LOC106764910, LOC106772003, LOC106773047, LOC106774971	Chr.1, Chr.2, Chr.4, Chr.5, Chr.6, Chr.8, Chr.9, Chr.10	Sequencing, Histogram plots, Statistical analysis, Molecular markers	(Sandhu and Singh, 2021)
AVRDC mini-core collection	297	:	MLM, GLM	5.3K	Seed coat luster	TASSEL 5.2.31, STRUCTUREv2.3.4, R-programming	*Vradi05g09110, Vradi05g09100, Vradi05g08320*	Chr. 5	Molecular Markers, statistical analysis, data integrations	(Breria et al., 2020b)
AVRDC mini-core collection	284	Controlled Conditions	FarmCPU, MLM	5.3K	Salinity stress	TASSEL 5.2.31, STRUCTUREv2.3.4, R-programming	*Vradi07g0163, Vradi09g09600, Vradi09g09510*	Chr.7, Chr.9	Molecular Markers, statistical analysis, data integrations	(Breria et al., 2020a)
USDA accessions	95	Summer	MLM, GLM	6.48k	Seed minerals Zn, P, S, Mn, K, Fe, Ca	TASSEL, BWA, R-programming	*Vradi01g00840, Vradi01g00830, Vradi01g00820, Vradi05g16350, Vradi07g26340, Vradi07g26320, Vradi07g1418*, Vradi*08g22740, Vradi06g10210, Vradi06g10120, Vradi06g10060, Vradi06g10020, Vradi06g09900, Vradi07g06200, Vradi07g05950, Vradi01g05570, Vradi06g02380*	Chr.1, Chr.5, Chr.7, Chr8, Chr.6	Statistical analysis, Molecular markers, Mapping, Data integrations	(Wu et al., 2020b)
Australian accessions including wild types	482	Summer	MLM	22.2K	Seed coat color	TASSEL, R-programming, DARwin v6.0	VrMYB113, Vrsf3′h1	Chr.4, Chr.5	Mapping, Data integrations, Statistical analysis	(Noble et al., 2018)
Other Species										
Lentil accessions from 60 countries	326	Winter	MLM	164.1K	Aphanomyces root rot index, Root dry weight, Shoot dry weight,	Haploview (v 4.2), Cartographer, BWA, SAMtools, Freebayes (v1.2), VCFtools, BEAGLE (v 3.3.2), R-programming	ABCA, PE, and CHI	Chr.2, Chr.4, Chr.5, Chr.7	qRT-PCR, QTLs Mapping, Molecular markers Statistical analysis,	(Ma et al., 2020)
ICARDA lentil accessions	176	Winter	GLM	22.5K	Days to first flower, Plant height, Seed per pod, days to maturity, harvest index	TASSEL, Freebayes, BamTools, Stacks, RAD-Tags, PGDSpider, STRUCTURE, UPGMA, NTSYS-PC program 2.02k, CDC Redberry	Marker trait associations (MTAs) SLCCHR3, SLCCHR5, SLCCHR6, SLCCHR7	Chr.2, Chr.3, Chr. 5, Chr. 6, Chr. 7	Molecular markers, PCR, Statistical analysis,	(Rajendran et al., 2021)
Diverse Lentil accessions	200	Winter	MLM	21.6K	Resistance to anthracnose race 1	VCFtools, MSTMap, ICIMapping, KnowPulse database, SNPRelate, STRUCTURE, Bayesian-model-based, GAPIT, R-programming	*Lcu.2RBY.3g006340, Lcu.2RBY.3g006380, Lcu.2RBY.3g005880, Lcu.2RBY.3g005310, Lcu.2RBY.3g006350 and MATs includes* Lcu.2RBY.Chr6.374326758, Lcu.2RBY.Chr5.437944230, Lcu.2RBY.Chr5.28637458, Lcu.2RBY.Chr4.442702133, Lcu.2RBY.Chr4.442702129	Chr.3, Chr.4, Chr.5, and Chr.6	GenBank, Molecular Markers, QTL mapping, statistical analysis	(Gela et al., 2021)
Lentil accessions	143	Winter	GEMMA	22.2K	Identification of pre-biotic carbohydrates, Total Starch, Resistant Starch, Stachyose+Raffinose, Sucrose, Fructose, Glucose and Mannitol	VCFtools, GAPIT, TASSEL, FarmCPU, VanRaden, PLINK, R-programming	*Lcu.2RBY.7g016860, Lcu.2RBY.7g016850, Lcu.2RBY.6g060190, Lcu.2RBY.6g015410, Lcu.2RBY.3g007570, Lcu.2RBY.2g028680, Lcu.2RBY.2g028670, Lcu.2RBY.2g028680, Lcu.2RBY.2g028670, Lcu.2RBY.1g069450,Lcu.2RBY.1g023480, Lcu.2RBY.7g048400, Lcu.2RBY.7g048380, Lcu.2RBY.7g048450, Lcu.2RBY.7g048410, Lcu.2RBY.7g048380, Lcu.2RBY.4g007850,Lcu.2RBY.6g015410, Lcu.2RBY.5g043890, Lcu.2RBY.4g045790,Lcu.2RBY.2g055260,Lcu.2RBY.1g020350, Lcu.2RBY.1g020320, Lcu.2RBY.6g040560,Lcu.2RBY.4g026570, Lcu.2RBY.3g057050*	Chr.1, Chr.2, Cr.3, Chr.4, Chr.5, Chr.6, Chr.7	Statistical analysis, Histograms, GBS and molecular markers, and chemical analytical techniques through advanced instruments	(Johnson et al., 2021)
Lentil accessions	118	Winter	MLM	3.2K	Resistance to Pea Aphid (PA resistance traits	STACKS v.2.0, BWA, SAMTOOLS v.0.1.19, BEAGLE v.3.3.2, FarmCPU, HAPLOVIEW v.4.2, R-programming	Marker trait associations (MTAs) including 7173_43, 7453_32, 5957_51, 5443_27, 5421_34, 3884_57, 4584_48, 3782_10, 2642_48, 3385_39	Chr.2, Chr.3, Chr.4, Chr.5	Statistical analysis, Association mapping, PCR, Molecular markers	(Das et al., 2022)
Common bean Spanish diverse panel	308	Spring	MLM	32.8K	Pod morphological and color characters	fastPHASE, Tassel, mrMLM, GAPIT,	Phvul.010G118700, Phvul.010G117200, Phvul.008G019500, Phvul.007G206200, Phvul.006G074600, Phvul.002G141800, Phvul.001G262600, Phvul.001G229900, Phvul.001G139100	Chr.1, Chr.2, Chr.4, Chr.6, Chr.7, Chr.8, Chr.10	Statistical analysis, QTL mapping, Molecular markers, Sequencing	(García-Fernández et al., 2021)
Faba bean accessions from ICARDA and other countries	140	Winter, Spring	GEMMA	10.8K	Herbicide tolerance traits include Plant height, seeds per plant, pods per plant, branches per plant, yield per plant, days to maturity, and days to 50% flowering	ADMIXTURE, TASSEL, Bowtie, GenStat, BLUP, R-programming	SNP trait association including *SNODE_7114_58 (gene*, MYB-related protein P-like)*, SNODE_559376_60 (gene* photosystem I core protein PsaA*), SNODE_4187_38 (gene*, malate dehydrogenase*), SNODE_3696_16 (gene*, Probable serine/threonine-protein kinase NAK*), SNODE_14298_44 (gene*, LRR receptor-like serine/threonine-protein kinase RCH1*), SCONTIG127798_41(gene*, acidic endochitinase*)*	——	Molecular markers, Statistical analysis	(Abou-Khater et al., 2022)
Faba bean accessions from ICARDA and other countries	134	Summer, Spring, Winter	GEMMA	10.8K	Heat resistance including, Plant height, seeds per plant, pods per plant, branches per plant, yield per plant, days to maturity, days to flowering, pollen germination, and 100 seed weight	TASSEL, Bowtie, ADMIXTURE, TASSEL, BLUP, R-programming	LOC11440721, LOC113783927, LOC11440721, LOC109335950, LOC11420332, LOC11420332, LOC11420332, LOC11430352, LOC101493666, LOC101512103, LOC114380151, LOC113847809, LOC109813943, LOC25500962, LOC101496898, LOC101496898, LOC112012620, LOC101492966, LOC101492966, LOC11425609, LOC109813943	——–	Molecular markers, Statistical analysis	(Maalouf et al., 2022)
Faba bean accessions	290	Winter	MLM, GLM	687	Frost resistance traits including AUSPC, LTAF, LCAF, FAC, FPC,	TASSEL, PowerMarker, QTL Network, STRUCTURE, R-programming	Marker trait associations (MTAs) including VF_MT3G086600, VF_MT2G027240, VF_MT4G125100, VF_MT4G127690, VF_MT5G026780, VF_MT4G007030, VF_MT5G005120, VF_MT5G033880, VF_MT7G090890, VF_MT7G084010, VF_MT5G046030	Chr.2, Chr.3, Chr.4, Chr.5, Chr.7	QTL mapping, PCR, Molecular markers, Statistical analysis	(Sallam et al., 2016)
Faba bean a inbred lines	189	Winter	MLM	2.54K	Convicine and vicine contents in seeds	TASSEL, WinISI II, R-programming	SNPs associations, Affx-1003954634, Affx-1003937842, Affx-309473691, Affx-308714105, Affx-309732154, Affx-308989324, Affx-309859410, Affx-309903736, Affx-308750155, Affx-310120776, Affx-309712729, Affx-310628027, Affx-308848038, Vf_Mt4g053880	Chr.1, Chr.2, Chr.3, Chr.4, Chr.5, Chr.6	Molecular markers, NIR, HPLC, Statistical analysis, Genetic map, QTLs	(Puspitasari et al., 2022)
Chickpea ICRISAT accessions	280	Winter	MLM	4.6K	Zn and Fe concentrations, Day to 50% flowering, Days to maturity, and 100 seed weight	TASSEL, GAPIT, Admixture, BLINK, STRUCTURE, STRUCTURE HARVESTER, BLUPs, FarmCPU	S6_7891103, S7_9379786, S4_4477846, S6_26554579, S4_31996956, S1_2001361, S1_2772537, S7_32973784	Chr.1, Chr.4, Chr.6, Chr.7	Molecular markers, Statistical analysis, Mapping,	(Srungarapu et al., 2022)
Chickpea Australian accessions	315	Winter	MLM	298K	Yield related traits	BLUE, GeneStat, Tassel, R-programming	Evaluated the genetic variability based on the number of SNPs per chromosome. No gene was reported.	26.7K SNPs on Chr.1, 18.1K on Chr.2, 13.6K on Chr.3, 52.3K on Chr.4, 27.8K on Chr. 5, 129.3K on Chr.6, 25K on Chr.7, 5.5K on Chr.8	Statistical analysis,	(Liu et al., 2021)
ICARDA Chickpea accessions	186	Winter	GEMMA	5.3K	Salinity stress	BLUEs, ADMIXTURE, R-programming	*RPLP0, EMB8-like*	Ca2, Ca4	Statistical analysis, Molecular markers, Cross-validations	(Ahmed et al., 2021)
Chickpea accessions	92	Winter	CMLM, EMMAX	16.59K	Zn and Fe concentration in seeds	GAPIT, STACKS v1.0, FASTQC v0.10.1, CGAP v1.0, SnpEff v3.1h, BiNGO plugin of Cytoscape V2.6, PAMLv4.8a, TASSEL v5.0, PowerMarker v3.51, MEGA v5.0, STRUCTURE v2.3.4, PLINK, MALDI-TOF	Ca03227, Ca03400, Ca24399, Ca22196, Ca09146, Ca03842, Ca00947, Ca12262. Ca09416, Ca27126, Ca19289, Ca06927	Chr.1, Chr.2, Chr.3, Chr.4, Chr.5, Chr.7	Association mapping, QTL Mapping, HPLC, Molecular markers, RT-PCR, Statistical analysis	(Upadhyaya et al., 2016)

In contrast, Ahmed et al. (2021), for the first time in chickpeas discovered *RPLP0 and EMB8-like candidate genes using GWAS associated with salinity stress* (Ahmed et al., 2021)*. Similarly*, Maalouf et al. (2022) discovered candidate genes (MYB-related P-like protein, PsaA, RCH1, NAK, and LRR) through GWAS in faba beans associated with herbicide tolerance. The successful above-mentioned examples of candidate gene identification through GWAS provide strong evidence that GWAS can rapidly detect hidden loci/genes associated with important plant traits and that can be effectively used to further strengthen the mung bean breeding program.

## 4 Recent advances in high-throughput phenotyping in GWAS

Domestication started many decades ago in response to feeding the large population and protecting plants from adverse climatic conditions. Domestication requires many years (about 6 to 7 years mostly) to develop a single crop variety. This challenge forced researchers to find new ways to speed up the process of crop improvement. Therefore, various techniques were successfully introduced to improve crops within a short duration and whole genome sequencing was one of those techniques. Since whole-genome sequencing has been achieved in several crops, functional genomics studies have stepped into the big-data and high-throughput phenomics era. In 1911, Wilhelm Johannsen characterized the word phenotype for the first time as “all type of organisms can be distinguished by direct inspection or with finer method of measurements or description” (Johannsen, 1911). Later, Davis in 1949 defined the word phenome as “the total of extra genic, non-auto-reproductive portions of the cell and represented the set of phenotypes” (Davis, 1949). Simply, crop phenomics can be defined as “the multi-disciplinary study of high throughput accurate acquisition and multi-dimensional analysis of phenotypes on a large scale through crop development” (Yang et al., 2020). Plant phenotype is influenced by genotype and environment (G x E) interactions. According to Mendelian genetics, in the presence of a dominant allele, the recessive allele will not be expressed. Additionally, if the allele expression is being influenced by environmental factors (soil, light, temperature, etc.) then the dominant trait may only emerge under certain environmental conditions. Thus, phenotype is the sum of three-dimensional (3D) spatiotemporal expression information resulting from interactions between environmental factors and genotype. However, the acquisition of phenotypic data is still a bottleneck limiting functional genomics studies (Deery et al., 2016). Traditional phenotypic approaches mostly depend on manual measurements, which are subjective, time-consuming, laborious, and hamper comprehensive phenotypic data from individuals within a large population. Additionally, errors are obvious in manual measurements, and therefore, data reliability and accuracy data cannot be guaranteed (Xiao et al., 2022). In addition to cost, manpower, and other related limitations, manual measurements can only be exploited for limited features during the critical stages of plant growth. Moreover, physical changes cannot be fully detected throughout a plant’s life cycle. The aforementioned shortcomings and limitations from traditional approaches can be overcome by exploiting high throughput phenotyping (HTP). HTP is emerging as an important tool for evaluating a plant’s phenotype. HTP approaches such as fluorescence imaging, hyperspectral imaging, visible light imaging, automation technology, machine vision and advanced sensors combined with advanced information technologies (ITs) and data extraction systems have enabled more accurate, rapid, and non-destructive measurements of physiological and morphological parameters. Each of the above-mentioned techniques has its advantages that allow reliability and accuracy in high throughput detection (Jiang et al., 2018; Narisetti et al., 2021; Sarkar et al., 2021).

HTP platforms integrate data acquisition equipment, a control terminal, and data analysis platforms. Firstly, in HTP, phenotypic data are collected via spectroscopy and non-invasive imaging techniques and then high-performance computational tools are adopted to rapidly analyze plant physiological state and other growth activities. In comparison to traditional phenotypic approaches, HTP offers simultaneous data acquisition of multiple traits and close observation of plant activities at different growth stages throughout the life cycle. Secondly, traditional approaches like visual scoring, are prone to subjective interpretation while trait characterization in HTP is more based on images or spectra which are more objective. Thirdly, HTP offers modeling-based non-destructive estimation of biochemical parameters, hence reducing laborious tasks and time. In the last few years, there have been major advances in HTP techniques to study different targets such as plant roots, leaves, shoots, seeds, cells, and canopy (Yang et al., 2020). For example, microscopic imaging and microcomputed tomography (m-CT) are used in the determination of tissue morphology (Zhang et al., 2021), cell growth rate (Gallegos et al., 2020), alterations in cell structure (Faulkner et al., 2017) and number of cells (Mele and Gargiulo, 2020). Moreover, visible light imaging and 3D graphics have intensively been used for characterization of seed morphological traits like germination rate (Ligterink and Hilhorst, 2017; Merieux et al., 2021), seed weight (Huang et al., 2022), growth and development (Margapuri et al., 2021), coleoptiles length (Zhang and Zhang, 2018) and seed color (Baek et al., 2020). Other physiological, morphological, and biochemical parameters have also been intensively studied through combined GWAS and HTP using time domain pulsed nuclear magnetic resonance (NMR) (Melchinger et al., 2018), Semantic Guided Interactive Object Segmentation (SGIOS) (Yuan et al., 2022), Graphical User Interface (GUI) (Yuan et al., 2022), Near-infrared spectroscopy (Jasinski et al., 2016; Anderson et al., 2019a), Deep convolutional neural networks (DCNNs) (Jiang et al., 2021), Hyper-spectral vegetation indices (VIs) (Koh et al., 2022), unmanned aerial vehicle (UAV) (Jiang et al., 2021), computed tomography (Guo et al., 2022) and multi-spectral or hyper-spectral images (Wu et al., 2021a; Correia et al., 2022). In-depth information on phenotyping techniques can be found here (Rahaman et al., 2015). Zhang and Zhang (2018), in their review, summarized the applications of recently developed imaging HTP techniques to study the pathological, physiological, and morphological traits of plants (Zhang and Zhang, 2018). Shakoor et al. (2017), provided a detailed review of HTP techniques (especially recently developed sensors) in accelerating plant breeding and disease assessments (Shakoor et al., 2017). Recently Liu et al. (2020), thoroughly reviewed hyper-spectral imaging and three dimensional (3D) techniques applications for plant phenotyping (Liu et al., 2020). Jang et al. (2020), in their review have focused on UAV applications in plant breeding and summarized the deployed sensors that can be mounted on UAV and their characteristics in detail (Jang et al., 2020).

Phenotypic data is one of the most important factors limiting GWAS power, inaccurate and non-reliable phenotypic data results in false associations. For example, imprecise phenotypic data greatly influence the true MAF present within a population, so that the identified SNPs cannot be linked to traits that are affected by these SNPs. Phenotypic data collected manually is always prone to error. Therefore, to minimize these errors, HTP techniques are combined with GWAS. The success of this combination can be gauged by the number of studies published in the last 4 years. HTP combination with GWAS has made it possible to study those plant traits that cannot be studied through physical phenotypic parameters e.g. I-traits (traits that can only be studied efficiently through images) (Wu et al., 2021a). Furthermore, this combination also improves the crop selection process and makes selection strategies tractable for plant breeders to increase the rate of genetic gain (Crain et al., 2018). Wu et al. (2021a), combined an HTP technique called Plant array, a lysimetric-based system developed by Halperin et al. (2017), which combines several factors to measure plant water relations during plant life cycle with GWAS to study the physiological parameters for drought stress in 106 accessions of cowpea (Halperin et al., 2017; Wu et al., 2021b). They identified a total of 20 SNPs out of which 14 were significantly associated with critical soil water content (θ_cri_) and 6 were significantly associated with the slope of transpiration rate declining (K_Tr_). The detected SNPs were distributed on 9 different chromosomes and accounted for 8.7 to 21% of phenotypic variation, indicating both stomatal closure speed and stomatal sensitivity to soil drought were controlled by multiple genes with moderate effects. Wu et al. (2021b) established a multi-optical HTP system based on X-ray computed tomography and hyper-spectral imaging combined with GWAS to study drought stress in 368 maize genotypes using an I-trait pipeline (Wu et al., 2021b). Their data revealed 4322 significant locus-trait associations, representing 1529 QTLs and 2318 candidate genes. They also reported two novel genes *ZmFAB_1_A* and *ZmcPGM_2_* associated with drought stress and 15 I-traits as potential markers for maize drought tolerance breeding. Crain et al. (2022) combined the unmanned aerial vehicle (UAV) HTP technique with GWAS to study the relationships between single plant and full plot yield in 340 wheat accessions using association mapping panel for full plot and single plant association mapping for single plants (Crain et al., 2022). UAV (equipped with a multi-spectral camera) was used to collect normalized difference vegetation index (NDVI) throughout seasons (2018-2019 and 2019-2020). According to their data, both single plant and full plot NDVI measurements (during the grain filling stage) were positively associated with grain yield. They identified SNPs on chromosome 7A and 2B significantly associated with spikelet and spike length, respectively, during the growing season 2018-2019 but with no associations for the same traits were identified in 2019-2020 growing season. Moreover, SNPs marker identified on chromosome 4B were significantly associated with plant height within the full plot association mapping panel in both seasons. However, no association was found for the same trait within single plant association mapping for a single plant. Furthermore, canopy reflectance spectrometry combined with GWAS in strawberries increased the selection efficiency of resistant lines against powdery mildew (Tapia et al., 2022). Aerial-based systems combined with GWAS have greatly facilitated the measurements of canopy traits such as canopy coverage and lodging to further facilitate the identification of novel QTLs associated with such traits. RGB (Red, Green, and Blue) imaging and GWAS combination have been successfully exploited in detecting the genetic architecture related to disease resistance. Silva et al. (2022) used a ground-based proximal sensing HTP platform in combination with a DJI quadcopter Matric-100 multi-spectral imaging camera to screen wheat genotypes against barley yellow dwarf disease (BYD) (Silva et al., 2022). GWAS analysis identified 16 significant SNPs marker associated with resistance to BYD distributed on chromosomes 5AS, 7AL, and 7DL. They also identified the *Bdv2* gene on chromosome 7AL as having a strong association with resistance to BYD. Xiao et al. (2022) provided a review of advances in HTP techniques and also summarized the combined applications of HTP and GWAS in different crops such as wheat, rice, barley, maize, soybean, and other species till 2020 (Xiao et al., 2022).

So far, no study has been reported on a combined analysis of HTP and GWAS in mung bean. This combination of HTP and GWAS in mung bean can be useful for studying novel traits such as i-traits associated with biotic and abiotic stresses (Guo et al., 2018). Such traits can only be efficiently measured or calculated through aerial or imaging techniques. X-ray computed tomography; multi-spectral imaging, spectroscopy, 3D structural analysis, and RGB imaging can be used in mung bean to study the physiological and biochemical activities under stressful conditions throughout the life cycle. Combining the aforementioned HTP techniques with GWAS can identify novel loci or genes associated with yield-related traits and resistance to biotic and abiotic stresses. HTP techniques can measure phenotypic traits more rapidly and accurately and also improve selection efficiency in mung bean breeding programs.

## 5 Connecting GWAS with genome editing

**Figure 5:**
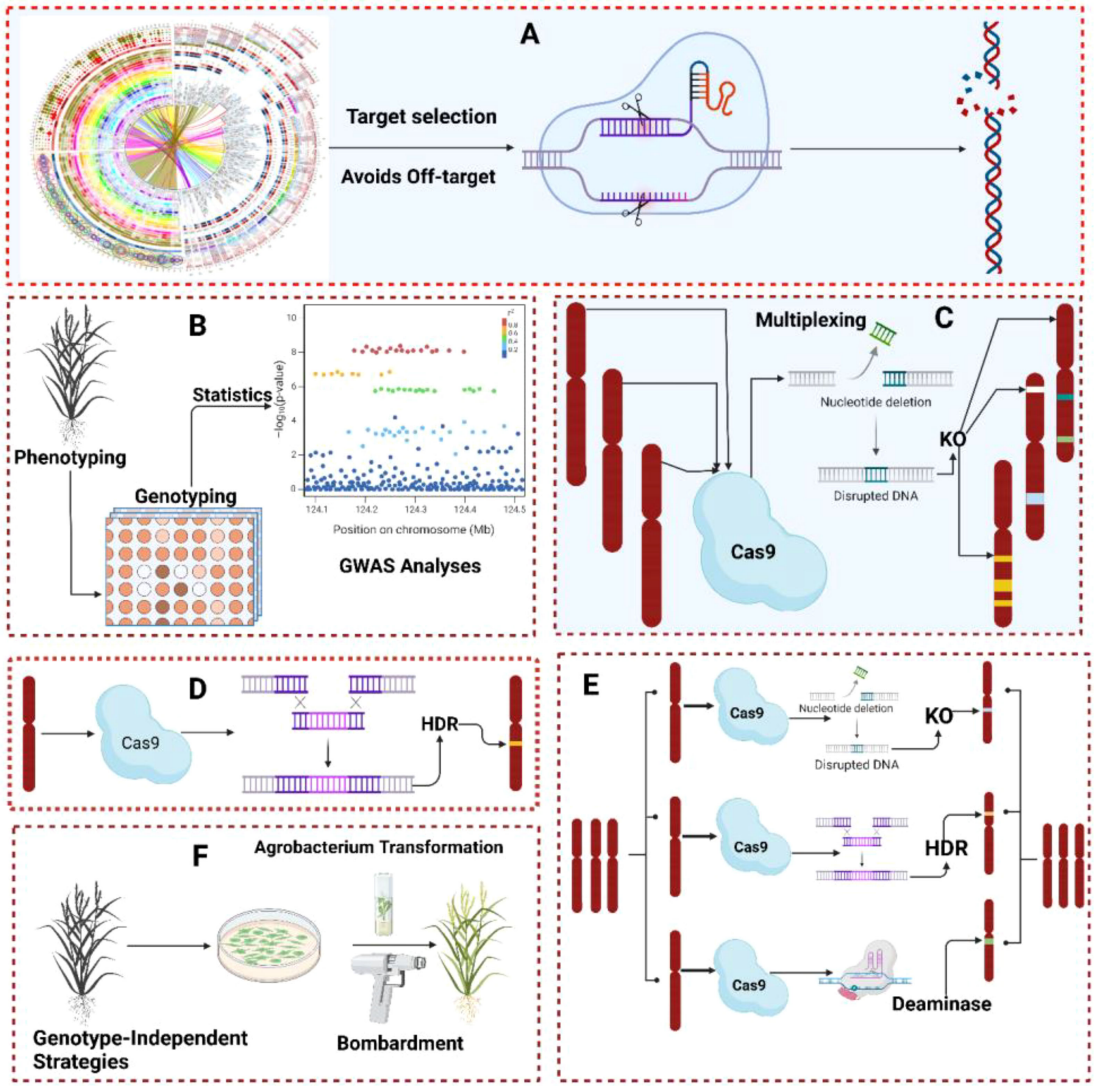
A simultaneous representation of GWAS and genome editing. **(A)** General overview of CRISPR-Cas from gene selection to genome editing. **(B)** Phenotyping, genotyping, and identification of the causal loci(s)/allele(s) associated with particulate trait. **(C)** Genome editing of loci/alleles identified by GWAS for further validation of results using gene knockout strategy **(D)** Genome editing of loci/alleles identified by GWAS for further validation of results using gene HDR and NHEJ strategy **(E)** Genome editing of loci/alleles identified by GWAS for further validation of results using gene KO, HDR, NHEJ and deaminase strategy **(F)** CRISPR-Cas most reliable delivery methods (Agrobacterium and Bombardment).

Genome editing (GE) technologies have revolutionized the field of life science by precisely editing plant genomes. In the past few years, different GE tools such as zinc finger nucleases (ZFNs), transcriptional activator-like effector nucleases (TALENs) and clustered regularly interspaced short palindromic repeat (CRISPR) have been successfully exploited for editing complex and simple plant traits. ZFNs are targetable DNA cleavage proteins that act as restriction enzymes to cut DNA sequences. ZFNs were artificially developed by fusing binding domains of ZFNs proteins with the Fok-1 endonuclease cleavage domain. Similarly, TALENs were also developed by fusing TALEs (transcription activator-like effectors) derived DNA binding domains with the Fok-1 endonuclease cleavage domain (Zhang et al., 2019). TALENs are capable of inducing double-stranded breaks (DSBs) in targeted sequences, which activates DNA repair pathways, resulting in genome modifications. However, both TALENs and ZFNs have been intensively used to edit the genome of living organisms including humans and plants, but some limitations of these technologies have prevented their effective use. Therefore, scientists started looking for other effective GE technologies and discovered the CRISPR-Cas9 system in archaea and bacteria (Jinek et al., 2012) (**Figure 5**). In the beginning, CRISPR-Cas also had limitations just like other GE technologies, but with time, different CRISPR-Cas variants were discovered to overcome these limitations. CjCas9 is a Cas9 variant, derived from *Campylobacter jejuni*, and is more specific in cutting targeted DNA sequences than Cas9 *in vivo* and *in vitro*. CjCas9 is delivered through AAV (adeno-associated virus) in the target cell and induces targeted mutations at high frequency (Kim et al., 2017). Recently discovered Cas13 is another variant that is used to target endogenous RNAs and viral RNAs in plant cells (Wolter and Puchta, 2018). Different research groups have reported that CRISPR-Cas13 is highly efficient and has the highest RNA target specificity compared with other Cas variants (Abudayyeh et al., 2017). NGS technologies have made precise target-specific gene editing much easier. Significantly associated SNPs controlling important traits have made CRISPR-Cas base editing more efficient than whole gene insertion and deletion. Combining GWAS and CRISPR-Cas system offers three key advantages; firstly, editing of identified SNPs/genes with CRISPR can further validate whether the identified SNPs/genes are indeed associated with trait of interest or not, secondly, putative genes with unknown functions identified through GWAS can be knocked-out to identify their functions, thirdly, insertion or deletion in candidate gene (identified through GWAS) can help in improving plant traits. For example, Kariyawasam et al. (2022) identified *SnTox5* (involved in facilitating *parastagonospora nodorum* colonization in mesophyll tissue of wheat to induce program cell death) gene using GWAS and edited through CRISPR-Cas system to further validate its previously reported role in pathogenesis. They identified *Sn2000_06735* (putative candidate gene) as a homolog of *SnTox5* and to validate this, *Sn2000_06735* was disrupted by inserting hyg^R^ (hygromycin resistance cassette) using the CRISPR-Cas system. *Sn2000_06735* disrupted mutants failed to cause necrosis and prevented *Parastagonospora nodorum* colonization (Kariyawasam et al., 2022).

Thus, confirming *Sn2000_06735* is associated with *Parastagonospora nodorum* pathogenesis. Similarly, Liu et al. (2021) identified the *Fov7* gene (encodes for GLR proteins) through GWAS that is associated with resistance to *Fusarium oxysporum* in *Gossypium hirsutum*. CRISPR-Cas system-based knockout of *Fov7* resulted in extreme susceptibility to *Fusarium oxysporum* in all-cotton lines. Moreover, they also identified the significant SNP in the *Fov7* gene associated with resistance to *Fusarium oxysporum* and revealed that this SNP changes an amino acid and confers resistance. Another group of researchers selected different rice cultivars using pedigree analysis to identify yield-related candidate genes through GWAS. They discovered six genes with known functions (associated with yield) and 123 loci with genes of unknown functions. From 123 loci, they randomly selected 57 genes for CRISPR-Cas-based system knock-out to identify their functions. Their results revealed that most of these genes were significantly associated with yield-related traits. For instance, *Os01g0885000*, *Os01g088600*, and *Os01g0555100* showed fewer tillers, a reduction in plant growth, and changes in panicle structure, respectively (Huang et al., 2018). Liang et al. (2022) phenotyped 2409 accessions of soybeans to identify the candidate gene involved in controlling the number of branches per plant (Liang et al., 2022). GWAS analysis revealed *SoyZH13_18g242900* (also known as *Dt2*) as a candidate gene significantly associated with the increase in number of branches per plant and several other agronomic traits. To validate the role of *SoyZH13_18g242900*, DN50 (soybean variety with four branches) was selected and *SoyZH13_18g242900*was knocked out using the CRISPR-Cas9 system. Field experiments revealed that *Dt2* mutant lines showed an increase in the number of branches compared with wild-type DN50. Moreover, these mutant lines also increased days to flowering and maturity and enhanced the number of nodes per plant and plant height.

## 6 Future prospects

### 6.1 Opportunities, challenges, and future strategies of GWAS and PWAS

The prior knowledge of natural genetic variations present in mung bean is extending and making mung bean a model crop to study genetic variations in other crops like mash bean, faba bean, and other pulses. We have observed these advancements in recent years through a large number of genetic variability studies conducted to understand the phenomena of natural variation in mung beans. GWAS soon will be more useful/informative in mung bean using advanced sequencing technologies to unlock the hidden genetic variations and availability of the high throughput SNPs set associated with phenotype as a reference genome in genebank e.g., IPK, to study the mutations in mung bean mutant genotypes and construct some useful genetic maps such as MutMap. The output of GWAS could be executed and utilized in different aspects, for example, improving breeding programs, targeted genome editing, identification of novel genes, constructing genetic maps, high throughput phenotyping or highly accurate phenotyping by breeders can also improve GWAS power in detecting new loci and recombinations. These advances help in facilitating and improving breeding by analyzing the genomics or genetics of agronomically important plant traits. In-depth analysis in detecting causative loci via GWAS, for instance, haplotype-based analysis is a key for genomics-assisted plant breeding. In comparison to QTLs mapping, GWAS has higher resolution due to the large number of recombination’s and large population comprising hundreds to thousands of genotypes used to study genetic variations in more depth and breadth. GWAS in future mung bean work must be considered as an exploratory analysis for selecting true segregating parents which can be utilized in developing populations and QTL mapping and in the future for molecular and genetic association validations. Besides, GWAS is also useful in understanding marker-based selection (individual selection for breeding programs based on their available genetic information of specific alleles linked to QTLs) or breeding-program-based variation (the genetic variability of association panel implemented in improving crops) because the association mapping population is considered as a source of alleles that are rarely present in bi-parental mapping populations. Recently, various studies used both association mapping and QTLs mapping to isolate or identify and validate the QTLs associated with traits of interest for example, brassica (He et al., 2017), maize (Zhao et al., 2018b) and faba bean (Sallam et al., 2016). This technique utilizes both populations (biparental and mixed population) to determine whether the identified significant markers are associated with the same trait of interest in two different genetic backgrounds or not. However, no study in mung bean has been reported yet using this technique and therefore, it will be of great advantage to implement it in mung bean to genetically improve the traits of interest. Association mapping population is always rich in alleles (including land races, wild types and domestication alleles) and offers great genetic variation; therefore, it can be considered as an excellent genetic resource and enhance the chances of discovering new genes/alleles controlling complex traits such as yield, tolerance to biotic and abiotic stresses. The analyses enable predicting the function(s) of the different alleles representing genetic alterations/mutations and candidate alleles/genes which are associated or have an agronomic impact, thus could be utilized in molecular validations such as genome editing and gene expression. Collaborations with bioinformaticians and statisticians can help in establishing new efficient statistical models and databases that can be utilized during the analysis of complex traits. Integration of genetics and omics can be crucial for molecular analysis. Therefore, they should be integrated and implemented together. The expansion in natural variation analysis to molecular mechanisms will further provide insights into mechanisms involved in mung bean growth, adaptation, and development.

The advancements in genomic approaches offer opportunities to characterize genetic diversity, traits mapping, and improvements and they also offer a greater understanding of complex genomes and the development of new genome editing tools for breeding.

#### 6.1.1 Complex polyploid genome, genetic resources, and rapid domestication of crop species

Autopolyploidy and allopolyploidy are common mechanisms of genome doubling and many plants (especially angiosperms) during evolution have undergone at least two rounds of polyploidy. This natural mechanism results in introducing more allelic diversity, improving crop adaptation to new environmental conditions and new phenotypic variations. Plant breeders have already taken several advantages of this mechanism by introducing artificial polyploids with an increase in fruit size (Wu et al., 2012), developing seedless fruits (Varoquaux et al., 2000), and increasing the grain yield (Rosyara et al., 2019). Genomic studies in polyploidy species have always been a great challenge due to several complications and reasons. Besides, the development of a genomic library with high quality, there is another challenge due to the inclusion of different but closely related sub-genomes, differentiating homologous loci and generating non-mosaic sub-genome scaffolds. Different research groups have made efforts to reduce the genomic complexity of polyploids by sequencing closely related species (Shulaev et al., 2011) or diploid progenitors (D’hont et al., 2012) to generate initial reliable reference assemblies. Detection of SVs and SNPs in closely related species is still very challenging and difficult and most of the studies have failed in detecting these variations (Gordon et al., 2020). Besides these difficulties, genetic improvement of polyploids is subject to further complications: (1) dissecting the genetic architecture of complex traits becomes impossible when the variants are not mapped to the correct sub-genome (Ramírez-González et al., 2018) and (2) biologically, the exact prediction of phenotype based on genotype might be hampered by extensive epistatic interactions and regulatory feedback between sub-genomes in polyploids (Bird et al., 2018). However, these issues have already been addressed through advancements in sequencing and assembly algorithms. As the numbers of GWAS and Pan-genomic studies are expanding in polyploid crop species, we expect that the degree of SNPs, *k-mers*, and SVs will be greater compared with diploid species.

Breeding efforts using pan-genomic studies are limited because only a few research groups are using this technique and therefore, the genomic resources remain low. For example, *Silphium integrifolium* (an oil crop species with large genome size) genome was studied using transcriptome assemblies to identify loci associated with adaptation in different climatic conditions due to the non-availability of whole genome reference genomic assembly (Raduski et al., 2021). SVs remained uncharacterized in this study due to limited genomic resources and SNPs helped in identifying the loci by re-sequencing. Forage crops and turfgrass are other examples of crops with limited genomic resources. GWAS and PWAS have unlocked the challenges associated with crop domestication, especially with the reduction in the time frame generally required for developing a single variety. Plant breeders can use genomic information resulting from GWAS/PWAS to genetically improve crops efficiently by genome editing techniques or identifying markers or variants (PAV, CNV, and SNPs) associated with particular traits in wild plants. For instance, pan-genomic in tomatoes revealed that variations in fruit size/weight are controlled by the duplication of the *SKILUH* (cytochrome P450) gene (Alonge et al., 2020), rather than an SNP as reported earlier (Chakrabarti et al., 2013). Later, this was confirmed by using CRISPR-Cas9 to reduce the *SKILUH* copy number, and resulted in alterations in fruit weight (Alonge et al., 2020). Domestication of crops has significantly reduced the genetic diversity compared with wild relatives. Identification and utilization of the genetic diversity from wild relatives is a major focus of a plant breeder in improving crops. Combined applications of GWAS and genome editing technologies will allow *de-novo* domestication of wild plants and take advantage of available genetic diversity from secondary and tertiary gene pools (wild plants). Wild relatives of mung bean are known to possess high genetic diversity. Therefore, domestication with wild relatives is easy as till now no study has reported the combining ability barriers between domesticated and wild parents. For instance, the mini core of mung bean from world vegetable gene bank Taiwan, studied GWAS in a large mung bean population (containing all the domesticated and wild relatives) to identify the SNPs associated with the trait of ineptest. They identified several SNPs associated with the trait of interest are in wild relatives rather than in the domesticated plants. The wild relatives had more SNPs and had more strong association with phenotypic traits (Sokolkova et al., 2020). This study is the proof that the domestication has reduced the genetic diversity in the mung bean to significant level. However, this can be restored by crossing the domesticated mung bean plants back to wild relatives.

### 6.2 Challenges, future applications, and role of high throughput phenotyping in GWAS

Various studies have demonstrated the potential applications and role of HTP in plant research but few studies have integrated GWAS and HTP. The key factors that limit GWAS and HTP integration are challenges in the accession of genomic data, accuracy in characterizing phenotypic traits, and shortage of skilled persons. Genomic data can be obtained from re-sequencing or the gene banks. Reduction in the cost of whole genome re-sequencing has made genomic studies easier but it is still time-consuming and highly laborious. On the other hand, the data available in the genebanks at the movement may not match the actual samples due shortage of genebanks. Highly accurate phenotypic data of any trait is necessary for GWAS but currently available HTP techniques applied in GWAS are still generally flawed. Many HTP techniques such as X-ray CT, hyper-spectral imaging, and visible light/RGB imaging, strongly rely on data/image processing algorithms. Recently, signal-based algorithms have been associated with several deficiencies like inaccurate feature extraction, imperfection, and low efficiency; therefore, they need to be subjected to required improvements. Highly sensitive and high-resolution equipment utilized for fluorescence imaging, X-ray CT, and hyper-spectral imaging are very expensive and therefore cannot be implemented extensively. UAVs for near-surface HTP are appropriate for collecting phenotypic canopy data in the field due to wide spatial coverage and flexibility. However, complex approaches, huge prices, and insufficient payload acquired for processing enormous remote sensing data may limit their adaptation. Besides these, there is a need to introduce more promising HTP techniques like optical coherence tomography and infrared-thermal imaging in GWAS. Currently, some efforts have already been taken to acquisition of highly accurate phenotypic data using currently available HTP techniques (Mochida et al., 2019; Liu et al., 2020; Yang et al., 2020). Changes in phenotypic data are due to alterations in genetic composition and environmental factors. Environmental changes are directly associated with changes in the phenotypic traits of plants which are difficult to control. Indeed, we can develop phenotypic databases through HTP techniques but only if we consider a wide range of environmental situations which is challenging.

To enhance the implementation of HTP in GWAS to explore the underlying complex genetic architecture of phenotypic traits in mung bean and other plant species, the following aspects must be considered,

1. Enhance the amount of investment in developing more efficient and highly accurate population genotypic data approaches.
2. Sufficient genotypic data of various crops including mung bean must be present in online databases (https://bigd.big.ac.cn/gvm/home) and must be accessed by all the researchers. Collection of plant material of known genotypes for HTP is one of the potential strategies to reduce the associated cost.
3. The development of efficient HTP techniques with low cost is another strategy to encourage the wider application and adaptation of this technique in GWAS.
4. The development of new and improvement of existing public phenotypic databases are of great interest to efficiently resolve resource issues in data provision, heterogeneous data formats, and insufficient meta-data. We strongly recommend and urge the publication of meta-data, that need to be structured according to the principles of FAIR (Wilkinson et al., 2016)and state the detailed information like environmental conditions and data formats. Ćwiek-Kupczyńska et al. (2016) have already proposed the guidelines for governing the description of phenotypic data, which provided a document of Minimum Information About a Plant Phenotyping Experiment(MIAPEE) and encouraged to implementation of ISA-Tab format for meta-data set organization (Ćwiek-Kupczyńska et al., 2016). Furthermore, we need to develop universal unified standard formats for phenotypic data recorded using different approaches. Some efforts are under the mission of generating efficient phenotypic databases like PHENOPSIS DB for *Arabidopsis thaliana* (http://bioweb.supagro.inra.fr/phenopsis/). This database can be used as a template to develop more phenotypic databases for other crops like mung bean including other pulses and cereals.
5. Incessant developments of imaging algorithms or multivariate data are essential. For example, image processing and voluminous data in-depth processing have shown excellent impact in understanding the data, owing to their unique strength in the form of self-learning ability and efficiency in large data analysis. We do not doubt that in the future the applications of in-depth learning considering plant traits data extraction will be a hot research topic.
6. A combination of all existing HTP techniques might greatly facilitate the evaluation of plant traits in different aspects.

We are urgently in of need a large number of studies implementing GWAS and HTP techniques together to study diverse plant populations and traits to understand functional genetics/genomes in greater depth.

## Data Availability

The original contributions presented in the study are included in the article/**Supplementary Material**. Further inquiries can be directed to the corresponding author.
